# *uhrf1* and *dnmt1* Loss Induces an Immune Response in Zebrafish Livers Due to Viral Mimicry by Transposable Elements

**DOI:** 10.3389/fimmu.2021.627926

**Published:** 2021-03-29

**Authors:** Elena Magnani, Filippo Macchi, Bhavani P. Madakashira, Chi Zhang, Fatima Alaydaroos, Kirsten C. Sadler

**Affiliations:** Program in Biology, New York University Abu Dhabi, Abu Dhabi, United Arab Emirates

**Keywords:** *uhrf1*, *dnmt1*, transposable element, interferon, TNFa, zebrafish, DNA methylation

## Abstract

Activation of transposable elements (TEs) can cause cellular damage. Cytoplasmic nucleic acid sensing pathways evolved to detect pathogens, but can also serve to cull cells with inappropriate TE activation as TEs can be viral mimetics. Epigenetic silencing of TEs is mediated in part by DNA methylation, but it is not clear if TE activation or the immune system contribute to the cellular damage caused by loss of DNA methylation. Here, we provide mechanistic insight into the observation of an activated interferon response in the liver of zebrafish larvae with deletion in critical components of the DNA methylation machinery, *uhrf1* and *dnmt1*. We focus on dissecting the relationship between DNA methylation, TE activation and induction of an immune response through cytoplasmic DNA and double stranded RNA sensing pathways and identify *tnfa* as a mediator of cell death in the liver of these mutants. Integrated RNAseq and methylome analysis identified LTR transposons as the most upregulated in these mutants and also the most methylated in control larvae, indicating a direct role of DNA methylation in suppressing this TE subclass. RNAseq analysis from these same samples revealed expression signatures of a type-I interferon response and of *tnfa* activation, mimicking the pattern of gene expression in virally infected cells. CRISPR/Cas9 mediated depletion of the cellular antiviral sensors *sting* and *mavs* reduced expression of interferon response genes and *tnfa* depletion dramatically reduced cell death in *uhrf1* mutant livers. This suggests that the antiviral response induced by DNA hypomethylation and TE activation in the liver is mediated by the signaling pathways activated by both cytoplasmic double stranded RNA and DNA and that *tnfa* mediates cell death as a potential mechanism to eliminate these damaged cells.

## Introduction

The primary functions of the immune system are to sense danger and to differentiate non-self from self in order to control the expansion of infectious agents. One way that danger is sensed by immune and non-immune cells alike is the presence of nucleic acids in cellular locations where they are not meant to be. For instance, the presence of DNA or double stranded RNA in the cytoplasm is monitored by cytoplasmic sensors. These sensors trigger signaling and transcriptional pathways that lead to release of interferons and cytokines that recruit immune cells, shut down viral reproduction, and promote cell death (1). In most cases, these responses clear infected cells and prevent invasion of the infectious agent. If the signal persists, however, chronic activation of anti-viral pathways can lead to pathologies, as exemplified in the hyperinflammation that characterizes autoimmune diseases (2) and, of more recent interest, the exacerbated immune response to SARS-CoV-2 infection which causes severe or lethal COVID-19 (3).

In addition to sensing pathogen invasion, cytoplasmic nucleic acid sensing pathways are also utilized in settings where cells are damaged, such as in response to toxic exposures, oncogenic transformation, genotoxic stress, or epigenetic damage (4). In these cases, damaged or dead cells release nucleic acids into the cytoplasm or extracellular space that are detected as inappropriate by the anti-viral sensing pathways and trigger sterile inflammation. In addition to cell damage, the expression of latent endogenous retroviruses (ERVs) and transposable elements (TEs) can activate the same mechanism to cause sterile inflammation. This can be advantageous, as cells with TE activation can be oncogenic (5), and therefore it is beneficial to have an immunomodulatory mechanism to eliminate potentially pre-cancerous cells.

It is not known how changes to the epigenome contribute to immune activation. One idea is that the chromatin landscape can restrict or potentiate the transcriptional activity of key transcription factors such as STAT1, NF-kB, interferon response factors (IRF) 3 and 7, and others (6) that mediate the response to cytoplasmic nucleic acid sensing pathways (7). Evidence for this model is suggested through studies on the regulation of Tumor Necrosis Factor alpha (Tnfa). This highly pleiotropic cytokine affects nearly all cell types to trigger cellular responses spanning from the induction of inflammatory pathways, stimulation of cellular proliferation and differentiation to the activation of programmed cell death such as apoptosis and necroptosis. Loss of DNA methylation causes robust induction of Tnfa in the gut of zebrafish larvae (8) and in mouse macrophages (9). This is accompanied by modest decrease in the level of DNA methylation in the Tnfa promoter, leading to the conclusion that DNA methylation directly represses the expression of the Tnfa gene. An alternative hypothesis is that widespread changes in the repressive epigenome can derepress TEs, mimicking a viral infection, activating an interferon response, and activation of Tnfa. In this model, DNA methylation loss directly impacts the expression of TEs, and Tnfa is activated by an indirect mechanism. Delineating between these mechanisms is an important step in determining how widespread changes to the methylome contribute to chronic immune activation. In addition, it is important to determine if there are tissue specific immune responses to DNA methylation loss, as most studies in the field are carried out using cell culture models.

We approached this through studying the effect of widespread loss of DNA methylation during development of zebrafish embryos by deleting *uhrf1*, a core component of the DNA methylation machinery. *uhrf1* recognizes hemi-methylated DNA and recruits DNA methyltransferase 1 (*dnmt1*) onto replication foci to maintain the DNA methylation pattern in daughter cells (10, 11). Unlike mouse embryos where *uhrf1* deletion is embryonic lethal (12), *uhrf1* mutant zebrafish embryos survive past the early embryonic stages due to maternal supplies (11–13). Loss of either *uhrf1* or *dnmt1* in zebrafish causes profound developmental defects leading to premature death by 8 days post fertilization (dpf) (13–16). *uhrf1* is essential for cell proliferation and development of the eye (17), intestine (8, 16), and liver (14, 15). Our previous work in *uhrf1* mutant zebrafish larvae showed increased circulation of neutrophils and activation of an interferon response, causes by cytoplasmic nucleic acid sensing pathways triggered by TEs expression (18). This is complemented by studies by others where loss of *uhrf1* is pro-inflammatory: *uhrf1* mutant zebrafish have intestinal inflammation associated with cell death that is dependent on *tnf* a (8), mice with *uhrf1* deficient regulatory T-cells develop spontaneous colitis in part due to a loss of the immunomodulatory function of these cells (19) and a recent study showing that deleting *uhrf1* in mature T-regulatory cells leads to spontaneous inflammation in multiple organs and the acquisition of a pro-inflammatory gene expression pattern, counteracting the immunosuppressive function (20). In addition, loss of *uhrf1* in macrophages makes mice more susceptible to colitis in response to stimuli and *uhrf1* deficient macrophages display an enhanced proinflammatory profile when stimulated (9). Given the implication of *uhrf1* in many immune mediated responses, it is important to identify the mechanism by which alterations in *uhrf1* expression or function are proinflammatory.

Here, we investigate specific outcomes of DNA methylation loss in zebrafish livers. We focus on the liver because inflammation plays a central role in several important liver diseases, including viral and non-viral hepatitis, which, in the setting of fatty liver can progress to a life threatening steatohepatitis (21) and the inflammation during hepatic fibrosis can severely reduce liver function and regenerative capacity (22). Some studies have uncovered inappropriate TE activation as a common feature of liver cancer (23, 24) and in other cancer types, viral mimicry by TEs has been proposed to trigger activation of the interferon response (25–27). In the current study, we test the hypothesis that the interferon response in the liver of zebrafish larvae with loss of DNA methylation is mediated by unleashed TEs that trigger cytoplasmic nucleic acid sensing pathways and we test whether Tnfa, a key downstream target of these pathways, is involved in the cell death phenotype that characterizes the hepatic phenotype of *uhrf1* mutants. We find that LTRs are preferentially induced in these models, and that these same TEs are heavily methylated in wild-type (WT) embryos. We uncover a robust type I interferon response, activation of NF-kB and Tnfa signaling in the liver, which was attenuated upon deletion of cytoplasmic viral sensors suggesting that the nucleic acid sensing pathways, and not direct epigenetic regulation of immune genes, trigger the immune response. Furthermore, we discovered that *tnfa* depletion rescues cell death in *uhrf1* mutant livers. This advances the understanding of how DNA hypomethylation leads to a tissue specific hyperactivation of inflammatory mechanisms and shows that the immune response contributes to the removal of cells with epigenetic damage.

## Materials and Methods

### Zebrafish Husbandry and Genotyping

Adult fish were raised in accordance with the policies of the NYU Abu Dhabi for Animal Care and Use Committee (IACUC) on a 14:10 h light:dark cycle at 28°C. *uhrf1* [hi272 allele; (28)] and *dnmt1* [s904 allele; (29)] mutant embryos were generated by incrossing of heterozygous carriers and were identified based on characteristic phenotypes of small liver, small and defective jaw, small eye, and flat gut as described (14, 15) or by genotyping individual embryos. *uhrf1*^−/+^ adults were genotyped by PCR as described (14) (see Supplementary Table 1) and *dnmt1*^−/+^ were identified by outcross to *dnmt1*^−/+^ adults. To be able to use the *Tg(c269*^°*ff*^*; 10XUAS:dsRed)* line to monitor DNA methylation in the liver of live larvae, we generated a line that expresses the Gal4 driver in hepatocytes *Tg(fabp10a:Gal4;cmlc2:EGFP)* (Supplementary Figure 1). This serves to activate transcription from an unmethylated 10XUAS:dsRed reporter which is included in this line, but cannot activate the 10XUAS:GFP in the *Tg(c269*^°*ff*^*; 10XUAS:dsRed)* line because that promoter is silenced due to accumulation of 5-methyl cytosine (5-MeC) (30–32). In *Tg(fabp10a:Gal4;cmlc2:EGFP; c269*^°*ff*^*; 10XUAS:dsRed)* larvae, EGFP will only be activated in the in hepatocytes when the promoter is unmethylated (Supplementary Figure 1), such as in *uhrf1* and *dnmt1* mutants.

The Tg(*fabp10a:Gal4*; cmlc2:EGFP) line was generated using Gateway cloning (Invitrogen) to produce vectors with *tol2* transposon sites flanking the transgenes (33). Transposase mRNA was produced by using mMessage mMachine kit (Invitrogen) by following manufacturer's instructions. 40 ng of vector containing the Tg(*fabp10a:Gal4*; cmlc2:EGFP) cassette was injected together with 80 ng of transposase mRNA. Larvae with *cmlc2:EGFP* expression were raised and outcrossed to identify founders, and stable transgenics from allele A were crossed to the *c269*^°*ff*^ background.

### Crispr/Cas9 Generation and T7 Endonuclease Assay

sgRNA for *mavs* was designed by using ChopChop (https://chopchop.cbu.uib.no/). sgRNA for *slc45a2* (gene involved in pigmentation), *sting* and *tnfa* were previously designed and validated (8). Genotyping primers were designed by Primer3 (https://bioinfo.ut.ee/primer3-0.4.0/) and validated in USCS Genome Browser (https://genome.ucsc.edu/cgi-bin/hgPcr). sgRNAs were produced by sgRNA IVT kit (Takara Bio) by following the manufacturer's instructions and RNA was isolated by Trizol (Invitrogen). sgRNAs were quantified by Qubit RNA BR kit and diluted at 50 ng/μl and stored as single use aliquots. The efficiency of each sgRNA was assessed by injecting WT embryos with equal volume of previously diluted nls-Cas9 protein (IDT; 0.5 μl of nls-Cas9 added with 9.5 μl of 20 mM HEPES; 150 mM KCI, pH 7.5) and sgRNA, incubated at 37°C for 5 min and then 1 nl was injected in 1–2 cell stage embryos. At 24–72 hpf, 12–16 embryos from each sgRNA were individually collected and genomic DNA was extracted by heat shock denaturation in 50 mM NaOH (95°C for 20 min). For each embryo, PCR was performed on genomic DNA by using Q5 High-Fidelity Taq Polymerase (New England Biolabs) followed by T7 endonuclease I assay (New England Biolabs) to detect mutations. For T7 endonuclease I assay, 10 μl of PCR product was incubated with 0.5 μl of T7e1 enzyme (New England Biolabs) for 30 min at 37°C. Digested and undigested fragments were run in parallel in 2% agarose gel to assess the presence of indels. Efficiency was calculated as the number of embryos that show a positive result based on T7e1 assay divided by the total number of embryos assayed for each sgRNA.

All sgRNAs demonstrated to generate indel mutations were injected into the 1-cell embryos generated by an incross of *uhrf1*^−/+^ adults as previously described. The resulting F_0_ larvae were considered crispants. For each clutch and each sgRNA, *uhrf1*^−/−^ mutants were divided from phenotypically WT siblings at 5 dpf based on morphological differences and used for following analysis.

### Terminal Deoxynucleotidyl Transferase dUTP Nick end Labeling Assay

Larvae collected at 5 dpf were fixed in 4% Paraformaldehyde for 4 h at room temperature, and gradually dehydrated through a graded series of methanol and stored in 100% methanol at 4°C overnight. Gradual rehydration to PBS through a graded series of methanol/PBS dilutions was carried out at room temperature. Larvae were permeabilized with 10 μg/ml Proteinase K (Macherey-Nagel) in PBS containing 0.1% tween (PBST) and fixed in 4% Paraformaldehyde for 10 min at room temperature. Livers were then dissected out of the larvae and subjected to TUNEL assay according to manufacturer's instructions (*In Situ* Cell Death Detection kit, Fluorescein; Roche). Nuclei were counterstained with Hoechst (Thermo Fisher Scientific) diluted 1:1000 in PBS, mounted on a microscope slide with Vectashield (Vector Laboratories) and covered with a 0.1 mM coverslip for imaging using Leica SP8 confocal microscope. LAS X software (Leica software) was used for quantification from 3 separate optical sections per livers which were then averaged from 3 livers per clutch per condition and 4 clutches per sample were analyzed. Results were plotted in GraphPad Prism 8.

### RNA and DNA Extraction

For each sample, 10 to 20 livers were microdissected and RNA was extracted using Trizol (Invitrogen) following the manufacturer's instructions with some modifications. Briefly, during precipitation in isopropanol, 10 μg of Glycoblue (Thermo Fisher Scientific) was added and precipitation was performed overnight at −20°C followed by 1 h centrifuge at 12,000 g at 4°C. RNA was resuspended in water and used in the following procedures. Genomic DNA was extracted from 20 to 30 livers by using a DNA extraction buffer (10 mM Tris-HCl pH9, 10 mM EDTA, 200 mM NaCl, 0.5% DSD, 200 μg/ml proteinase K) as previously described (18). DNA was resuspended in water and quantified by Qubit dsDNA High Sensitivity kit.

### cDNA Production and qPCR

After RNA extraction, RNA was retrotranscribed without quantification. cDNA was synthetized using Qscript cDNA synthesis kit (Quanta Bio) following the manufacturer's instructions. cDNA was diluted 12 times and used for qPCR using Maxima® SYBR green/ROX master mix (Thermo Fisher Scientific). *rplp0* was used to normalize expression levels by using the calculations for delta-Ct and WT siblings were used to calculate delta-delta-Ct (DDCt) as previously described (34). To determine changes in expression between control and experimental samples, the fold change was calculated, the log_2_ was derived (L2FC) for display. All experiments were performed on samples from at least 3 independent clutches as indicated in the figure legends.

### Slot Blot Analysis of 5-MeC

Slot blot was performed as previously described (15). Briefly, 2 ng of genomic DNA was denatured in 400 mM NaOH/10 mM EDTA and blotted onto nitrocellulose membrane in duplicate for dsDNA and 5-MeC using a slot blot apparatus. Membranes were incubated 1 h at 80°C, blocked with 5% skim milk in TBST (37 mM NaCl, 20 mM Tris pH 7.5, 0.1% Tween 20), and incubated overnight at 4°C in either anti-dsDNA (Abcam, 1:8000 in 2% BSA in TBST) or anti-5-methyl-cytosine (m5C–Aviva Biosystem clone 33D3, 1:2000 in 2% BSA in TBST). Membranes were washed in TBST and probed with anti-mouse HRP secondary antibody (Promega; 1:5000 in 2% BSA in TBST) for 1 h at room temperature followed by development in ECL (Thermo Fisher Scientific). ChemiDoc (BioRad) was used to detect and quantify the chemiluminescent signal. Gel Analyzer was used to measure the signals and ratio between 5-MeC and dsDNA was plotted for controls and mutants in each clutch.

### RNAseq

Total RNA was extracted from ~20 livers dissected from 5 dpf zebrafish larvae for each condition. For *uhrf1*^−/−^ mutants and their phenotypically WT siblings, 5 clutches were collected while for *dnmt1*^−/−^ mutants and their phenotypically WT siblings, 3 clutches were used. RNA was treated by DNAse I for 30 min at 37° C followed by RNA purification (RapidOut DNA Removal Kit–Thermo Fisher Scientific). RiboZero was used to remove ribosomal RNA and the remaining sample was used for library preparation according to manufacturer's instructions (Illumina) from 80 to 100 ng of RNA. Libraries were sequenced on NextSeq550 (Illumina) to obtain 150 bp paired-end reads. Sequencing quality was assessed by using MultiQC v1.7 (https://multiqc.info). Adaptor sequences were removed and reads were aligned to the *D. rerio* GRCz10 reference genome using HISTA2 for alignment with default parameters so that only paired reads were aligned and multiple alignments were kept (35). To estimate gene expression, reads that mapped to the exon of each gene that had an annotated Ensembl ID were counted with HTSeq (36). A generalized linear model implemented in DESeq2 in Bioconductor (37) was adopted to test differential gene expression between each mutant compared to their respective sibling controls. Adjusted *p*-value with a false discovery rate of <0.05 was treated as significantly different expression between mutant and controls. Data is available in GEO (GSE160728).

TE quantification was assessed using RepeatMasker based on the annotation of *danRer10* provided by the UCSC Table Browser (https://genome.ucsc.edu/cgi-bin/hgTables) and were counted with HTSeq using union mode which allowed for each read to be counted only once; reads were not designated as strand specific. All TEs were quantified based on families. Statistical analysis was implemented with DESeq2 using the same protocol as the differential gene expression analysis described above for TEs with simple repeats excluded.

### Reduced-Representative Bisulfite Sequencing

RRBS was performed on genomic DNA extracted from 10 *uhrf1*^−/−^ mutants and phenotypically WT siblings at 5 dpf. Briefly, 50–250 ng of gDNA was digested with 200 U of MspI (New England Biolabs) for 24 h at 37°C. Digested DNA was used for preparing library as previously described (38), with the exception that the adaptors used for multiplexing were purchased separately (Next Multiplex Methylated Adaptors–New England Biolabs). Libraries were size-selected by dual-step purification with Ampure XP Magnetic Beads (Beckman Coulter, Agencourt) to specifically select a region of fragments from 175 to 670 bp. Bisulfite conversion was performed with Lightning Methylation Kit (ZYMO Research) by following the manufacturer's instructions. Libraries were amplified using KAPA HiFi HotStart Uracil+ Taq polymerase (Roche) and purified with Ampure XP Magnetic Beads (Beckman Coulter, Agencourt) before sequencing. Libraries were sequenced using the Illumina Nextseq550. Fastq files are available in GEO (GSE160728).

Quality control of the RRBS sequencing data was assessed using FASTQC (http://www.bioinformatics.babraham.ac.uk/projects/fastqc). Reads were quality trimmed using Trimmomatic (39) to remove low quality reads and adapters. Reads passing quality control were aligned to the genome reference GRCz10 using the default parameters in Bismark (40), which adopts Bowtie2 as the aligner (41) and call cytosines methylation at the same time.

CpG methylation levels were detected with the R package “methylKit” (42). CpGs covered at least 10 times in each condition were included in the analysis. CpGs with methylation level below 20% were treated as unmethylated and above 80% were considered as methylated. Genomic element annotation of CpGs was performed with R package “genomation.” For plotting and statistical analysis, R package “ggplot” and GraphPad Prism 8 software were used. Transposons were identified using the Repeat Masker table annotation on the reference genome assembly GRCz10 (danRer10). The WashU EpiGenome Browser (http://epigenomegateway.wustl.edu/browser/) was used to display distinct genome locations.

### Bioinformatics

RNAseq and RRBS data were analyzed as previously described and visualized in RStudio (version 4.0) using code that is publicly available on Github (https://github.com/zcmit/NYUAD_Sadler-Lab/blob/master/uhrf1%20and%20dnmt1%20loss%20induces%20an%20immune%20response%20in%20zebrafish%20livers%20due%20to%20viral%20mimicry%20by%20transposable%20elements). For Gene Ontology (GO), zebrafish gene names were converted in human gene names by using Biomart and then used for the GO analysis. GO enrichment analysis was conducted using the GO hypergeometric over-representation test in the “ClusterProfiler” package in R using default parameters, and REVIGO was subsequently used to eliminate redundant enriched terms. An adjusted *p* < 0.05 was treated as significant for all analyses. Specific gene lists of type-I interferon response and Tnfa were collected from IPA database and used to subset specific group of genes. Heatmaps were performed by using R package “pheatmap.”

### Statistical Analysis

All experiments were carried out on embryos from at least 3 biological replicates and, where appropriate, technical replicates were also included and are indicated in the figure legend of the relevant data. The number of replicates for each experiment is indicated in each figure. Methods to evaluate the statistical significance include Students *t*-test with adjustment for multiple comparisons or Chi square analysis; the tests used are indicated in each graph and table. All the plots were generated in GraphPad Prism 8 and RStudio. Statistical analysis is performed in GraphPad Prism 8.

## Results

### *uhrf1* and *dnmt1* Loss Causes DNA hypomethylation in the Liver

**Figure 1:**
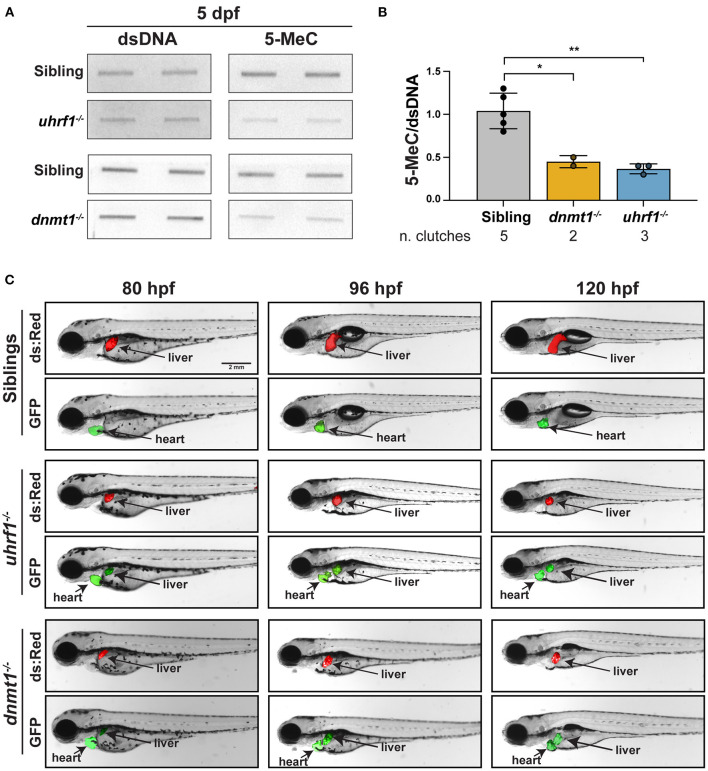
*uhrf1* and *dnmt1* loss causes DNA methylation loss in zebrafish livers. **(A)** Slot blot of genomic DNA extracted from pools of 5 dpf *uhrf1*^−/−^ and *dnmt1*^−/−^ mutant and WT siblings livers. **(B)** Quantification of 5-MeC measured by slot blot normalized to double stranded DNA (dsDNA). Each dot represents one clutch. *p < 0.05 and **p < 0.005 calculated by unpaired *t*-test. **(C)** Time course imaging of live larvae by fluorescent stereoscope microscopy. *Tg(c269*°*ff ; 10XUAS:dsRed; fabp10a:GAL4; cmlc2:EGFP);dnmt1*−/− and *Tg(c269*°*ff; 10XUAS:dsRed; fabp10a:GAL4; cmlc2:EGFP);uhrf1*−/− are shown at each age, showing the liver (dsRed), the heart (GFP) as a marker of transgenesis and, in green, the liver with DNA hypomethylation in mutants only. Images are representative of 100% of larvae from 3 clutches observed for each genotype.

We used a biochemical assay and a novel imaging approach that uses a methylation reporter in live zebrafish to assess the status of DNA methylation in the liver of *uhrf1* and *dnmt1* mutant larvae. First, bulk DNA methylation was assessed using slot blot analysis of 5-MeC on total genomic DNA extracted from the liver of 5 dpf larvae with mutation in *dnmt1* or *uhrf1* (Figures 1A,B). In both cases, 5-MeC is decreased by more than 50% compared to levels detected in the liver of phenotypically WT siblings, with equivalent levels of residual methylation in both models (Figure 1B). This is comparable to the degree of hypomethylation detected in whole larvae from these two mutants (13, 15).

We next used a novel *in vivo* DNA methylation reporter line based on the GAL4-UAS system [(43); Supplementary Figure 1]. In the line termed *c269*^°*ff*^, 10 copies of the UAS promoter lie upstream of GFP, and these have been silenced by DNA methylation over generations of breeding [Supplementary Figure 1, (30–32)]. This high level of methylation on the promoter blocks the ability of GAL4 to activate GFP expression in WT animals, but when the 10XUAS is unmethylated due to loss of *uhrf1* or *dnmt1*, GAL4 can access the promoter and GFP is expressed (Supplementary Figure 1). As a positive control for GAL4 activity, this line was also crossed to a line containing *Tg(10XUAS:dsRed)* in which the UAS promoter was not silenced by DNA methylation (30, 32), and therefore the expression of dsRed is used as a control for GAL4 activity. To monitor DNA methylation specifically in hepatocytes, we generated a transgenic line expressing GAL4 under the *fabp10a* promoter which utilized the *cmlc2:EGFP* cassette as a reporter of transgenesis *Tg(fabp10a:GAL4; cmlc2:EGFP)*. These transgenes were crossed to the *dnmt1*^+/−^ and *uhrf1*^+/−^ mutants to generate and *Tg(c269*^°*ff*^*; 10XUAS:dsRed; fabp10a:GAL4; cmlc2:EGFP);dnmt1*^+/−^ larvae. In phenotypically WT siblings, GFP was not detected in the liver of any larvae, whereas in the liver of all *uhrf1* and *dnmt1* mutants examined, GFP was detected as early as 80 h post fertilization (hpf), which is the time when their liver bud is easily visible using these transgenes, and GFP expression persisted through 120 hpf (Figure 1C). These data indicate that DNA hypomethylation in the liver of *uhrf1* and *dnmt1* mutants is detected as soon as hepatocytes differentiate. Previous studies showed that the small liver phenotype observed in these mutants is correlated with massive cell death (14, 15, 29). We hypothesize that the cell death and small liver phenotypes are due to both a direct effect of DNA methylation on the ability of hepatocytes to appropriately go through DNA replication and also to the activation of the immune system which could serve to induce cell death.

### Retrotransposons Are Overexpressed in the Liver of *dnmt1* and *uhrf1* Mutant Larvae

In terminally differentiated tissues, DNA methylation functions primarily in imprinting, maintaining chromosome stability and silencing repetitive elements such as TEs and pericentromeric DNA. More than 50% of zebrafish genome is constituted by repetitive elements, with DNA transposons making up the large majority of these (Supplementary Figure 2). Our previous analysis of TE expression in RNAseq generated from the whole *uhrf1* and *dnmt1* mutant larvae uncovered widespread retrotransposon activation (18).

**Figure 2:**
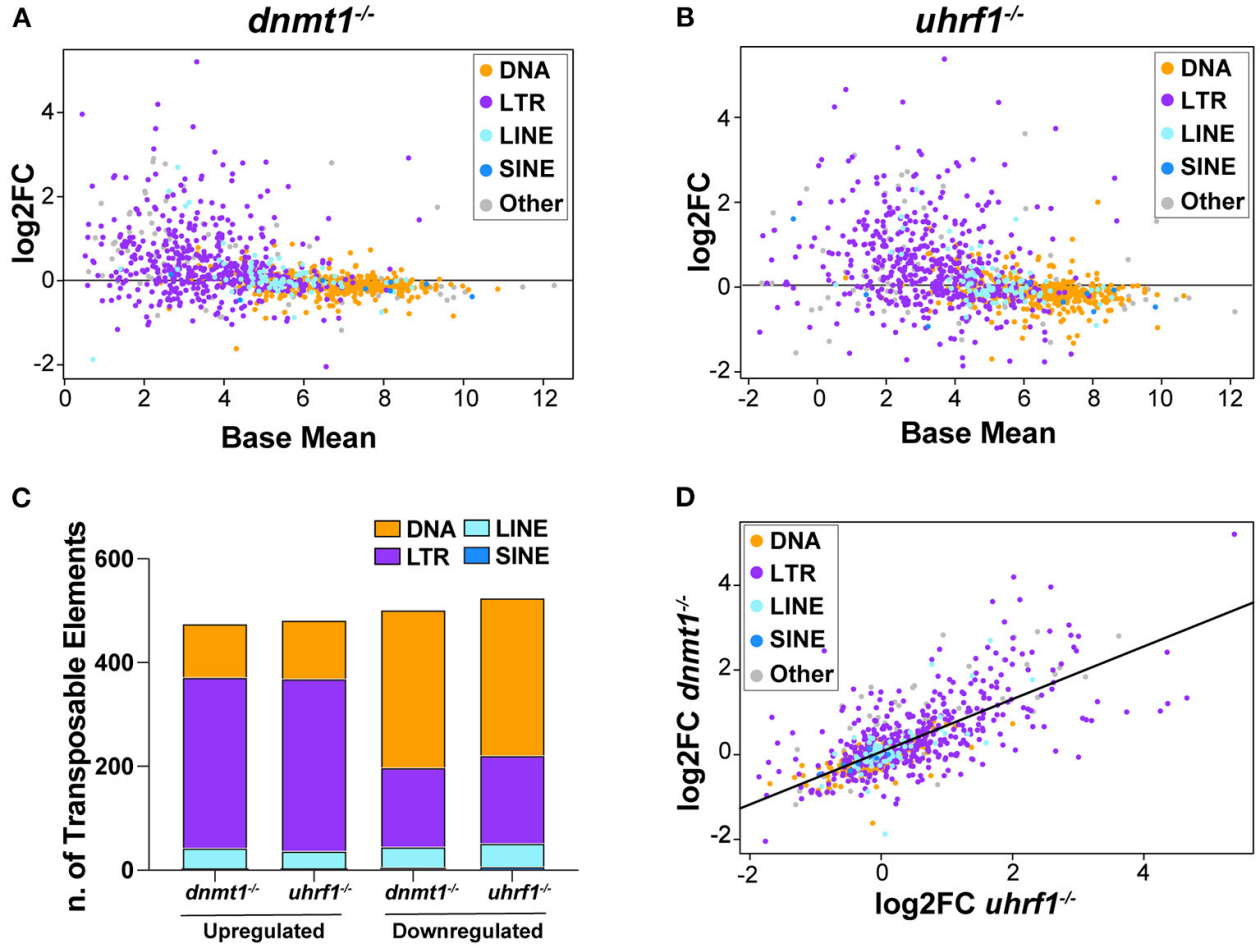
*dnmt1* and *uhrf1* loss causes overexpression of retrotransposons. RNAseq analysis of Transposable Elements in *uhrf1*^−/−^ and *dnmt1*^−/−^ mutant livers. **(A)** MA plot showing log_2_ fold change of repetitive elements in *dnmt1*^−/−^ livers calculated on WT siblings and Base Mean in WT siblings. Repetitive elements are divided by Class in DNA transposons (yellow), LTR (purple), LINE (light blue), SINE (blue), and other (gray). **(B)** MA plot showing log_2_ fold change of repetitive elements in *uhrf1*^−/−^ livers calculated on WT siblings and Base Mean in WT siblings. Repetitive elements are divided by Class in DNA transposons (yellow), LTR (purple), LINE (light blue), SINE (blue), and other (gray). **(C)** Bar graph of Transposable Elements divided by class in *uhrf1*^−/−^ and *dnmt1*^−/−^ mutant livers. Upregulated TEs have log_2_ fold change > 0 and downregulated TEs have log_2_ fold change < 0. **(D)** Correlation plot of repetitive elements in *uhrf1*^−/−^ and *dnmt1*^−/−^ mutant livers. Upregulated TEs have adj as pedix < 0.05 and log_2_ fold change > 0; downregulated TEs have *p*_*adj*_ < 0.05 and log_2_ fold change < 0. Log_2_ fold change is calculated between mutants and their own WT siblings.

Given that tissue specific expression patterns of both the genes that regulate TE expression and the TEs themselves have been reported (44), we used RNAseq to ask whether there was specificity in the TE expression pattern in the liver of 5 dpf *dnmt1* and *uhrf1* mutants compared to phenotypically WT siblings. A caveat is that the repetitive nature of TEs means that the short-reads generated by next generation sequencing could fail to capture the full spectrum of expression. Regardless, this method uncovered widespread changes in TE expression in both models. LTR transposons predominated as the most affected with 329 and 332 LTRs categorized as upregulated in *dnmt1* and *uhrf1* mutant livers, respectively (Figures 2A–C, Table 1). This is an overrepresentation, as LTR transposons occupy < 10% of the zebrafish genome (Supplementary Figure 2). Among the LTRs, members of the Gypsy and Pao families were the most upregulated and the most enriched in the datasets from *uhrf1* and *dnmt1* mutants (Supplementary Table 2). In contrast, while the DNA transposons dominate the TE landscape in the zebrafish genome (Supplementary Figure 2), only 103 and 113 DNA transposons were upregulated in *dnmt1* and *uhrf1* mutants, respectively (Figure 2C; Table 1). The changes in the expression of some LTRs was dramatic, with log_2_ fold change (L2FC) ranging from >6 to < –2. In contrast, DNA transposons had a L2FC range ± 1 (Figures 2A,B,D). TE expression was highly correlated in both mutants, with LTRs showing the strongest linear correlation (Figure 2D). In addition to the upregulated TEs, there were 502 and 525 repetitive elements which were categorized as down-regulated in *dnmt1* and *uhrf1* mutants, respectively (Table 1), albeit modestly compared to the upregulated TEs (Figures 2A,B).

**Table 1:** Transposable elements are differentially expressed in *dnmt1*^−/−^ and *uhrf1*^−/−^ mutant livers at 5 dpf.

	***dnmt1^s904^***		***uhrf1^hi272^***	
	**Up**	**Down**	**Up**	**Down**
DNA	103^*^	304^*^	113^*^	304^*^
**Retrotransposon**				
LTR	329^*^	153^*^	332^*^	169^*^
LINE	41	39	35	45
SINE	2	6	2	7

* *p < 0.0001 measured by Chi-square test*.

One possible explanation for the differential expression of TEs in these models is that they are bystanders which are influenced by the transcriptional changes at neighboring genes. Alternatively, the unmasking of TEs could lead to the aberrant expression of nearby genes. Bioinformatic approaches to analyze short read sequences combined with the intrinsic nature of repetitive elements does not allow us to determine the precise genomic location of all reads derived from TEs. We therefore took an alternative approach to determine whether the upregulation of TEs were correlated with upregulation of neighboring genes, which could suggest a bystander effect. We selected 10 of the most upregulated TEs in *uhrf1* mutants (Supplementary Table 2) and then counted the number of copies of each transposon in the zebrafish genome (Supplementary Figure 3A). We then identified the nearest gene, with no distance limit, to each location and determined the expression (log base mean) of that gene in the RNAseq datasets from *uhrf1* mutant and WT sibling livers (Supplementary Figure 3B). Note that in some cases, two or more TEs of the same family were in close proximity so that a single gene was assigned as the nearest neighbor of multiple TEs. If these upregulated TEs are coregulated with neighboring genes, then these genes should also be expressed at higher levels in *uhrf1* mutants. We found that only the genes that were detected close to “Gypsy105-I_Dr” and “Gypsy153-I_Dr” to be significantly upregulated in *uhrf1* mutants compared to WT siblings while there was no correlation between expression of TEs and expression of genes in their proximity (Supplementary Figure 3B) for all the other analyzed transposons.

Together, these results correlate loss of DNA methylation in zebrafish livers with derepression of expression of TEs, in particular of LTRs. Moreover, these data suggest that DNA methylation does not exert a uniform essential repressive function on all classes of TEs, as not all classes are found to be differentially expressed in these models. Finally, these data indicate that the expression of most TEs in these datasets is not attributed to a bystander approach, indicating a direct and inverse relationship between LTR expression and DNA methylation.

### TEs Derepressed in Hypomethylated Livers Are Heavily Methylated in Controls

**Figure 3:**
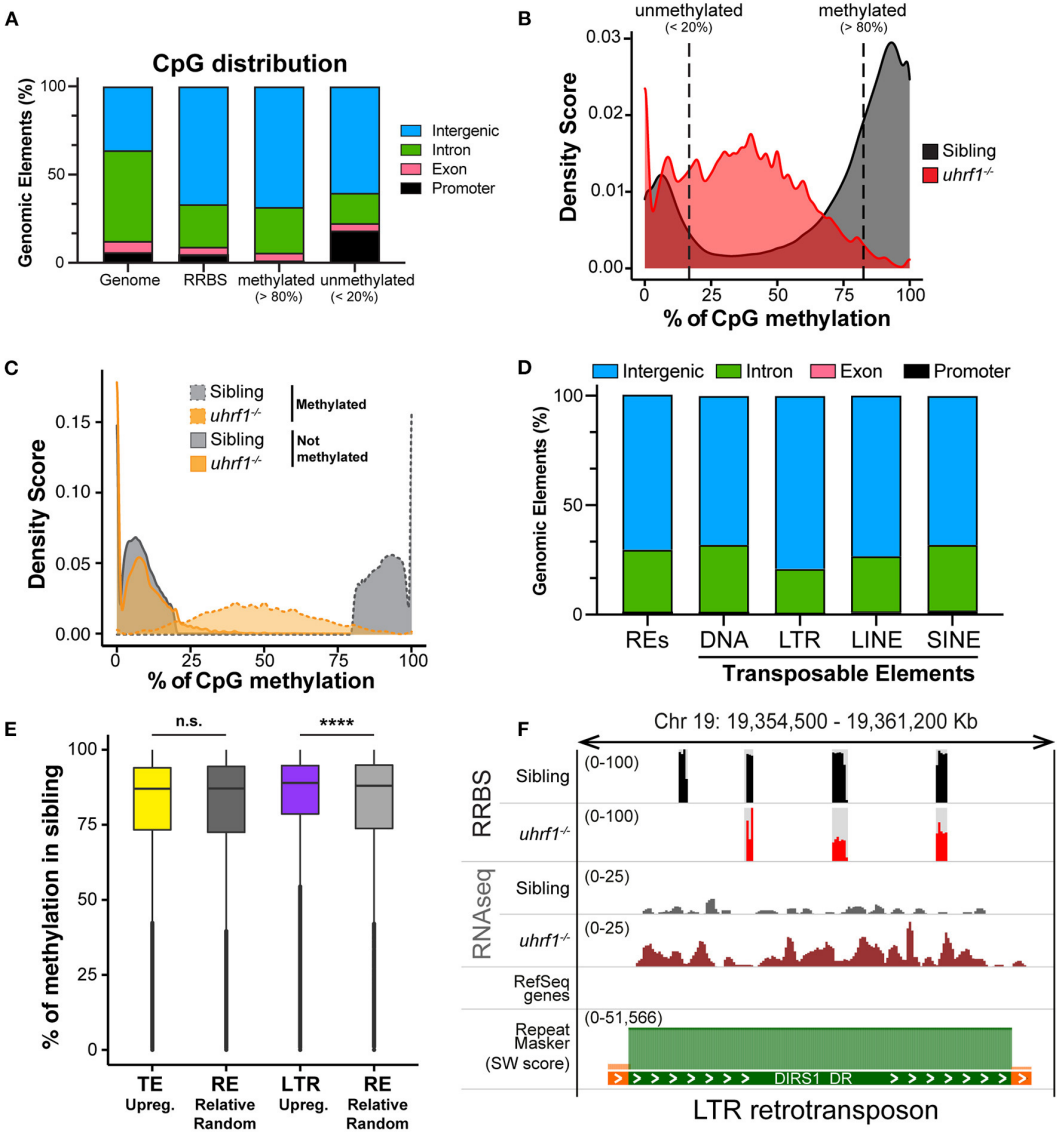
DNA methylation is enriched on TEs that become activated in *uhrf1* mutants. RRBS analysis on genomic DNA of TEs in *uhrf1*^−/−^ mutant larvae. **(A)** Genomic Annotation of all CpGs common to the unified dataset from *uhrf1*^−/−^ mutants and WT siblings are divided by level of methylation in WT siblings in methylated (>80%; 616,305 CpGs) and unmethylated (< 20%; 212,750 of CpGs) and were then classified based on their location in annotated genomic element. **(B)** Density plot of percentage of methylation of CpGs in *uhrf1*^−/−^ mutants and wild-type siblings. **(C)** Density plot CpGs in *uhrf1*^−/−^ mutants and wild-type sibling. CpGs were classified based on the percentage of methylation in the sibling in methylated (>80%) (dashed gray line) and not methylated (< 20%) (solid gray line). For each group, methylation levels were plotted for both mutants (dashed orange line) and siblings (solid orange line). **(D)** Genomic annotation of CpGs covered in RRBS and overlapping with the TEs annotated in the RNAseq. **(E)** Box plot describing the percentage of methylation of CpGs in WT siblings: from left, CpGs contained in TEs upregulated (*p*_*adj*_ < 0.05 and log_2_ fold change > 0 in *uhrf1*^−/−^mutants–yellow) and in equal number of REs randomly selected (193,397 regions–dark gray); CpGs contained in LTRs upregulated (*p*_adj_ < 0.05 and log_2_ fold change > 0 in *uhrf1*^−/−^mutants–purple) and in equal number of REs randomly selected (30,353 regions–gray). ****p < 0.0001 calculated by unpaired non-parametric Mann–Whitney test. **(F)** Genome browser screenshot shows an example of RNA transposons (LTR retrotransposon, DIRS1_DR) that is demethylated and expressed in *uhrf1*^−/−^ mutants. SW score is determined by Repeat Masker and it is used as indicator of the age of transposons. High SW score corresponds to highly conserved TEs, indicating younger TE.

In order to further delineate the relationship between DNA methylation and TE expression in this system, we performed Reduced-Representative Bisulfite Sequencing (RRBS) on two biological replicate samples of DNA extracted from whole 5 dpf *uhrf1* mutant and WT sibling larvae. We combined these two replicates to increase genome coverage so that our dataset captured 4.75% of the all CpGs present in the zebrafish genome (Table 2). This method enriched for CpGs in the intergenic regions and reduced coverage of CpGs in introns (Figure 3A) and therefore largely captures the TEs which are found in the intergenic regions (Supplementary Figure 4). To determine the pattern of DNA methylation changes between WT and mutant samples, all CpGs that were common to both datasets were categorized as methylated if >80% of the reads indicated 5-MeC, and unmethylated if this score was < 20% of reads. As expected, most of the methylated CpGs were found in the intergenic regions whereas the unmethylated CpGs are enriched in promoters and depleted from introns in WT siblings (Figure 3A). Also as expected, there is a bi-modal distribution of CpG methylation in WT larvae, with most of the CpGs in either methylated or non-methylated state (Figure 3B; Table 3). This is consistent with the finding that in terminally differentiated tissues CpGs can be either in a methylated or unmethylated state and bulk level of DNA methylation is between 75 and 85% (45, 46). In *uhrf1* mutants, this bi-modal pattern is lost (Figure 3B), as all the fully methylated CpGs are lost and the average level of methylation shifts to around 35% (Figure 3B; Table 3), consistent with the level of bulk DNA methylation loss detected by slot blot [Figure 1A, (15)]. We found that the CpGs which were fully methylated in control samples shifted to partial methylation of 45% (Figure 3C), while CpGs that were unmethylated in controls remained unmethylated in *uhrf1* mutants. This emergence of a large population of partially methylated CpGs likely reflects the heterogenous cell population analyzed in these samples extracted from whole embryos.

**Table 2:** RRBS analysis of *uhrf*1 whole embryos at 5 dpf.

	**Sequenced reads**	**Mapping rate %**	**CpG_**10**_ (coverage >10)**	**% of CpGs covered %**	**CpG_**10**_ common to all samples**	**#Common methylated CpG_**10**_**	**#Common unmethylated CpG_**10**_**
Sibling #1	15,468,377	43.6	1,565,271	6.53	1,138,340 (4.75%)	616,305	212,750
Sibling #2	15,662,370	42	1,567,074	6.54			
*uhrf1^−/−^*mutant #1	16,023,806	36.9	843,023	3.52		33,064	319,740
*uhrf1^−/−^* mutant #2	24,087,844	35.6	1,166,438	4.87			

**Table 3:** RNAseq analysis of *uhrf1* and *dnmt1* mutant livers at 5 dpf.

	***uhrf1^**−/−**^* mutant**	***dnmt1^**−/−**^* mutant**
Total number of genes detected	19,464	23,413
Significant genes (*p_*adj*_* < 0.05)	7,587	5,397
Significant UP (*p_*adj*_* < 0.05 & log_2_FC > 1.5)	2,595	1,578
Significant DOWN (*p_*adj*_* < 0.05 & log_2_FC < −1.5)	756	679
Significant in both	3,908	
Significant UP in both	1,166	
Significant DOWN in both	252	

We next evaluated whether the pattern of methylation on TEs correlated with differences of their expression in *uhrf1* mutants by overlapping the RRBS with the RNAseq expression data. The CpGs that were both located in annotated TEs and also covered by our RRBS analysis were mainly present in the intergenic regions (Figure 3D), and reflects the distribution of the TEs in the genome (Supplementary Figure 4). We next tested the hypothesis that the TEs that are the most upregulated in *uhrf1* mutants would be those that were the most heavily decorated with 5-MeC in controls. Since the analysis of the expression of TEs is based on families, and for each TE there are multiple copies in the genome (Supplementary Figure 3A) is not possible to determine the precise genomic location that accounts for each TE read. We therefore analyzed the methylation status of all the possible locations in the genome for each TE family that was upregulated in *uhrf1* mutants and compared them to equal number of randomly selected repetitive elements and then assessed the level of methylation across these two aggregates of genomic loci. This showed that the baseline level of DNA methylation was uniformly high and not significantly different between the TEs that were upregulated in *uhrf1* mutants compared to random selected repetitive elements. In contrast, the upregulated LTRs have higher DNA methylation levels compared to equal number of randomly selected regions (Figure 3E). These data suggest that the LTRs which derepressed in *uhrf1* mutants are heavily methylated. This is exemplified by the LTR transposon DIRS1, which is heavily methylated in WT embryos and becomes unmethylated and upregulated in *uhrf1* mutants (Figure 3F). On the contrary, several DNA transposons do not change methylation upon *uhrf1* loss: DNA25TWA1_DR is not methylated in controls or mutants and remains silenced as well as TDR13B which is highly methylated in WT samples and, surprisingly, retains DNA methylation in *uhrf1* mutants, and is not expressed in either samples (Supplementary Figures 5A,B). These data underscore the pivotal role of DNA methylation in silencing retrotransposons in somatic tissues, and presents a more complex picture of how other TEs are suppressed.

### *dnmt1* and *uhrf1* Mutation Activates Anti-Viral Response in the Liver

**Figure 4:**
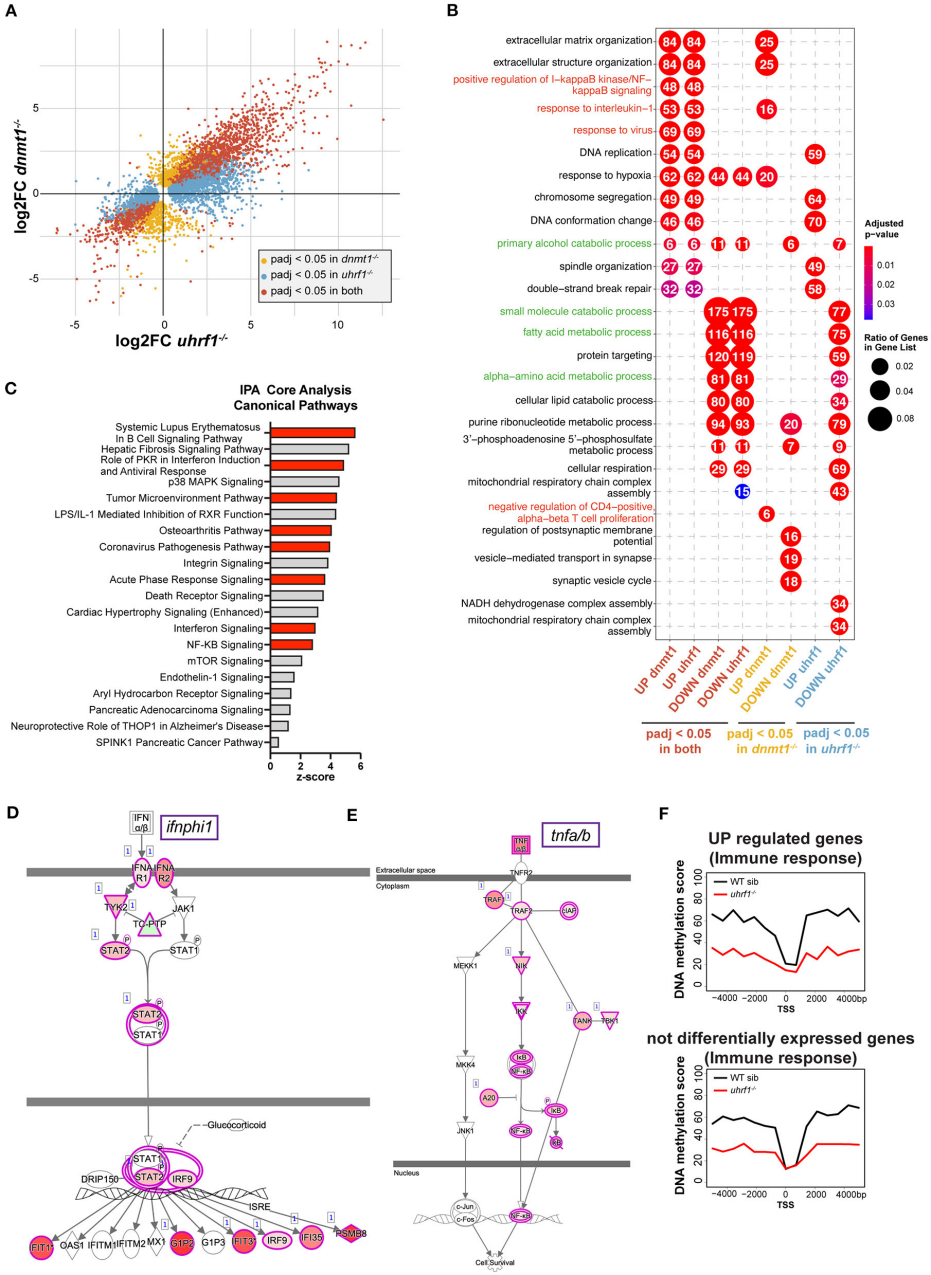
*dnmt1* and *uhrf1* loss activates typeI interferon and NF-kB mediated immune response. **(A)** RNAseq analysis of RNA extracted from *uhrf1*^−/−^ and *dnmt1*^−/−^ mutant livers. Four quadrants plot of log_2_ fold change of *uhrf1*^−/−^ and *dnmt1*^−/−^ mutant livers calculated on their own WT siblings. The genes that are significant are indicated in red. Genes that are significant (*p*_*adj*_ < 0.05) in *uhrf1*^−/−^ and *dnmt1*^−/−^, in yellow the genes significant only in *dnmt1*^−/−^ mutants and in blue the one significant only in the *uhrf1*^−/−^ mutant livers. **(B)** Gene Ontology of the upregulated and downregulated genes in each dataset. Significant genes (*p*_*adj*_ < 0.05) for each category in **(A)**, are divided based on log_2_ fold change in upregulated (log_2_ Fold Change > 0) and downregulated (log_2_ Fold Change < 0). In red the GO terms involved in immune pathways and in green GO terms associated to metabolism and liver specific pathways. **(C)** Bar graph shows the 20 most significant positively induced pathways according identified in IPA. Pathways are ranked ordered based on z-score and it indicates the likelihood of activation based on comparison with a model that assigns random regulation directions. In red, the immune related pathways. (**D)** IPA analysis of Interferon pathway. The color of the circles represents the expected trend of the genes when the pathways is upregulated (pink for upregulated and green for downregulated), the color inside the circles represents the observed trend of that gene in our RNAseq. **(E)** IPA analysis of Tnfa pathway. The color of the circles represents the expected trend of the genes when the pathways is upregulated (pink for upregulated and green for downregulated), the color inside the circles represents the observed trend of that gene in our RNAseq. **(F)** Metaplot of the DNA methylation levels in WT siblings and *uhrf1*^−/−^ mutants of the +/– 4 kb region surrounding the Transcription Start Site of the genes involved in Immune response pathways (GP006955) that are upregulated in the *uhrf1*^−/−^(*p*_*adj*_ < 0.05 and log_2_FC > 0, top panel) or not differentially expressed (*p*_*adj*_ > 0.05, bottom panel).

*uhrf1* or *dnmt1* deficiency has been shown to be a potent activator of a type I interferon response in tissue culture cells and in whole zebrafish larvae (18, 25, 27, 47). We used RNAseq to determine if this same pattern occurred in the liver of 5 dpf *uhrf1* and *dnmt1* mutants (Supplementary Figures 6A–D). In *dnmt1* mutant livers, 5,397 genes were significantly differentially expressed genes (DEGs; *p*_adj_ < 0.05); of these, 1,578 were upregulated [log_2_ fold change (L2FC) > 1.5] and 679 were downregulated (L2FC < −1.5) (Table 3). In *uhrf1* mutant livers, 7,587 DEGs were detected (*p*_adj_ < 0.05), with 2,595 upregulated (L2FC > 1.5) and 756 DEGs downregulated (L2FC < −1.5) (Table 3). This shows that the pattern of DEGs in both samples is toward the upregulation of genes, and also shows that many genes are highly induced in the liver of these mutants (Supplementary Figures 6A,B). While there are unique cellular functions of *uhrf1* and *dnmt1* which could induce distinct transcriptional responses, we reasoned that the responses induced by loss of DNA methylation would be shared in both datasets. Comparison of significant DEGs from both samples in a 4-quadrant plot shows a high correlation between the DEGs in these datasets (Figure 4A; Table 3), with < 8 of genes displaying a discordant expression pattern.

To determine the unique and shared cellular pathways that are differentially affected in the liver due to mutation of *uhrf1* and *dnmt1*, the zebrafish gene name was converted to the human gene name, and we then performed gene ontology (GO; Figure 4B) and Ingenuity Pathway Analysis (IPA; Figure 4C) on the human gene names, as annotation of human gene function is superior to the annotation of the zebrafish genome. We found that metabolic pathways and liver related processes are most represented among the downregulated pathways in both mutants (Figure 4B, in green; Supplementary Figure 7A), suggesting that hepatocytes do not achieve their full metabolic function in these mutants. In contrast, the majority of the upregulated pathways were immune related (Figures 4B,C). Of particular interest are the upregulation of the protein kinase R pathway and the corona virus pathogenesis pathway, which are induced in response to RNA viruses, NF-kB pathway, interferon signaling and response to virus. All of these are triggered by a nucleic acid sensing pathway that culminates in type I interferon response. One of the type I interferons, *ifnphi1*, is upregulated in both samples, whereas *ifnphi2* and *3* are only induced in *dnmt1* mutant livers (Supplementary Figure 7B). To further analyze the immune pathways deregulated in these samples we used IPA. This showed that the most prominent pathways activated the liver of these mutants are the pathways activated in autoimmunity (systemic lupus erythematosus), immune response to infection with an RNA virus (i.e., Coronavirus Pathogenesis Pathway), NF-kB and the interferon response (Figures 4C–E) among the most significantly enriched in the upregulated genes. The finding that the pathway activated by SARS-CoV2, an RNA virus which induces a distinct set of genes in the airway epithelial cells in infected patients (48) had significant overlap with the genes activated in *uhrf1* and *dnmt1* mutant livers (Supplementary Figure 7C) indicates that loss of DNA methylation induces an immune response similar to infection with an RNA virus (Figure 4C, Supplementary Figure 7C). ClueGO analysis provided a network view of the deregulated pathways, further showing that immune system processes were at the center of coregulated pathways in *uhrf1* and *dnmt1* mutant livers (Supplementary Figure 8).

To investigate the relationship between upregulation of immune genes and loss of DNA methylation on promoters, we analyzed DNA methylation levels around the Transcription Start Site (TSS) of upregulated immune genes in *uhrf1* mutants compared to WT siblings. While this shows that there is a significant loss of DNA methylation at these gene promoters, the same degree of loss is also seen around the TSS of immune genes that do not change expression upon *uhrf1* loss (Figure 4F). Since both groups of genes show a similar DNA methylation profile in WT and *uhrf1* mutant larvae, the upregulation of immune genes cannot be solely attributed to DNA methylation loss on their promoters. Since *uhrf1* mutation causes global loss of DNA methylation, this pattern was observed in all genes, and therefore it is not possible to rule out the possibility that, for some genes, DNA hypomethylation of promoters may impact expression. Regardless, these results are not consistent with the hypothesis that the induction of these genes is a direct effect of DNA hypomethylation in *dnmt1* and *uhrf1* mutants, but instead that the robust anti-viral immune response in the liver is attributed to the expression of TEs that are normally silenced by DNA methylation.

### Anti-Viral Signaling Components Sting and Tnfa Are Partially Required for the *uhrf1* Phenotype

**Figure 5:**
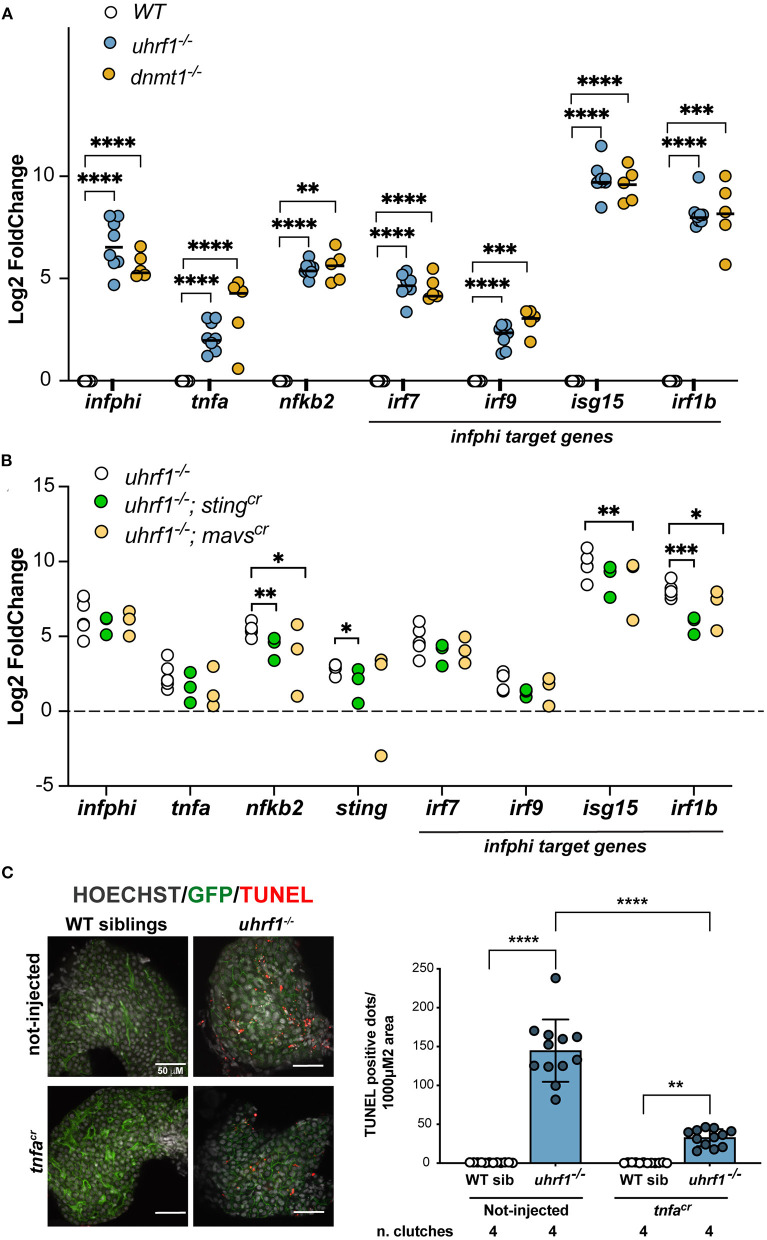
Viral signaling pathways are required for the gene expression and cell death phenotypes in *uhrf1* mutants. (**A)** qPCR analysis of immune genes in *uhrf1*^−/−^ and *dnmt1*^−/−^ mutants compared to their WT siblings. *Rplp0* is used as loading controls and the delta-delta Ct (DDCt) values were calculated by normalization to *rplp0* and WT sibling controls for each individual clutch. Lines in the graph represents the median. Statistical significance is calculated by paired t-test. **p < 0.05, ***p < 0.005, ****p < 0.001. **(B)** qPCR performed on the livers from *mavs*^*cr*^ and *sting*^*cr*^ F_0_ crispants in the *uhrf1* and WT backgrounds to assess immune gene expression. DDCt is calculated after normalization of each gene to *rplp0* and WT sibling controls for each clutch; this was performed for each crispants and for the not-injected embryos. Significance is calculated using 2-way Anova. *p < 0.05, **p < 0.01, ***p < 0.0005. **(C)** TUNEL analysis of *uhrf1*^−/−^ and phenotypically WT sibling livers at 5 dpf in *tnfa*^*cr*^ F_0_ crispants and non-injected controls. Quantification of the number of TUNEL positive foci per total liver area for at least 3 livers per clutch in 4 clutches for each condition. Significance is measured by 2-way Anova test. **p < 0.05, ****p < 0.0001.

To determine whether the antiviral response and the hepatic phenotype in *uhrf1* and *dnmt1* mutant larval livers was induced by cytoplasmic viral sensors, we used CRISPR/Cas9 to deplete essential genes regulating these pathways asking if this rescued the activation of immune response using qPCR. We selected a panel of genes involved in the antiviral response and we tested their expression in *dnmt1* and *uhrf1* mutant livers. *ifnphi1, tnfa*, and *nfkb2*, and interferon I target genes, such as *irf7, irf9, isg15*, and *irf1b* (Figure 5A) involved in this response were significantly upregulated in both *dnmt1* and *uhrf1* mutant livers (Figure 5A). Since the GO and IPA analysis highlighted the response to RNA viruses as activated and our previous studies suggested that the response to cytoplasmic DNA was involved in the immune response in *urhf1* mutants, we assessed key components of both pathways. Mavs is involved in detecting cytoplasmic double stranded RNA and Sting is activated by cytoplasmic DNA. Both sensors lead to activation of a type-I interferon response (49) and to induction of *tnfa*, a central signaling molecule that functions to trigger an anti-viral response.

sgRNAs were first validated for the ability to generate indel mutations when injected into WT and *uhrf1* mutant embryos (Supplementary Figure 9A). sgRNAs demonstrated over 90% efficiency when injected with Cas9 protein into 1 cell embryos generated from an incross of *uhrf1*^+/−^ adults. The F_0_ “crispants” were assessed for morphological abnormalities from 0 to 5 dpf and, at day 5, the left liver lobe size was measured to test whether the depletion of these genes influenced liver development. There were no significant changes in larvae morphology or liver size of the crispants in WT siblings or *uhrf1* mutants (Supplementary Figures 9B,C). At 5 dpf, livers were dissected and the expression of genes involved in the antiviral response were analyzed by qPCR in the liver of *uhrf1* mutant and phenotypically WT siblings. Although the statistical significance was reached only on some of the analyzed genes (*isg15, irf1b*, and *nfkb2*), all genes analyzed showed a decreased expression in the crispants compared to not-injected larvae (Figure 5B). This suggests that, overall, both *mavs* and *sting* are required for the expression of immune genes in *uhrf1* mutant livers.

Cytoplasmic nucleic acid sensing pathways lead to activation of the *tnfa* pathway (50). Tnfa can trigger apoptosis and necroptosis of infected cells (51, 52). Since *tnfa* signaling was activated in the liver of *uhrf1* mutants (Figures 4D,E) and deletion of *sting* and *mavs* significantly decreases *nfkb2* (Figure 5B), one of the main targets of *tnfa* cascade (Figure 4E), we hypothesized that Tnfa could mediate the cell death phenotype that characterized *uhrf1* (14, 15). To test this, Tnfa was depleted using CRISPR/Cas9 (Supplementary Figure 9A) in *uhrf1* mutants and used the TUNEL assay as a readout of the effect on cell death. While *uhrf1* mutant livers were characterized by high levels TUNEL positivity, in WT siblings TUNEL staining was completely absent (Figure 5C). *uhrf1* mutants that were *tnfa* crispants showed a significant decrease in TUNEL positive cells (Figure 5C). This indicates that activation of *tnfa* contributes significantly to cell death in *uhrf1* mutant livers.

These findings indicate that both the double stranded RNA sensing arm mediated by *mavs* and the cytoplasmic DNA sensing arm mediated by *sting* are activated upon DNA methylation loss and that they induce apoptosis that can be rescued, at least partially, by the deletion of *tnfa* indicating that the hyperactivation of this pathway is deleterious for the liver leading to cell death. Interestingly, the reduction of cell death did not rescue the small liver size in *uhrf1* mutants/*tnfa* crispants (Supplementary Figure 9C), suggesting that the cell cycle block in *uhrf1* deficient hepatocytes (14, 15) is the prominent driver of the small for size liver phenotype. Alternatively, it is possible that the TUNEL positive cells detected in the liver of *uhrf1* mutants could be detecting cell fragments in immune cells or even dying immune cells which infiltrate the liver in this model (18).

## Discussion

DNA methylation is a primary epigenetic modification that maintains repetitive regions of the genome in a repressed state and loss of the DNA methylation machinery during cell division leads to DNA hypomethylation. Many studies have demonstrated that DNA hypomethylation leads to cell damage and, in some cases, activation of an antiviral response. However, the relationship between DNA methylation loss, immune activation, and cell damage and death have not been fully investigated in whole animal models. Here, we investigated how DNA hypomethylation in the liver of *dnmt1* and *uhrf1* zebrafish mutant embryos leads to an immune response. Loss of *uhrf1* has been implicated in inflammatory bowel disease, based on findings that *uhrf1* zebrafish mutants develop inflammation in the intestine (8, 9, 16, 20) and in mice, *uhrf1* deletion in macrophages makes them hypersensitive to activation in the intestine (9). We hypothesized that depleting DNA methylation by *uhrf1* or *dnmt1* mutation would activate TE expression and triggering an antiviral immune response. Several studies, including ours, have demonstrated that loss of DNA methylation leads to activation of anti-viral pathways, in part, due to viral mimicry achieved by retrotransposons. We report that *uhrf1* or *dnmt1* mutation causes DNA hypomethylation in the liver and is associated with activation of a specific class of TE, LTR retrotransposons.

The immune response in cells with high level of aberrant expression of repetitive sequences has been coined as Transcription of Repeats Activates Interferons (TRAIN) by one group who reported that loss of DNA methylation and p53 inactivation, features common to most cancer cells, cause robust expression of TEs (47). We reported a similar response in whole zebrafish embryos with DNA hypomethylation due to mutation of *uhrf1* or *dnmt1* (18). Here, we expand on this finding to investigate the relationship between DNA methylation and TE activation in this model and then delineate the immune response in the liver of these mutants. This is particularly relevant to the liver, where inflammation is a critical factor in the progression of liver injury to liver disease and chronic inflammation is fundamental to the formation of cirrhosis and liver cancer.

Several studies performed in zebrafish, human cells and mice are consistent with our finding that DNA methylation loss activates RNA transposons, ERVs in particular (18, 27, 53–55). We report that TEs with high level of methylation are more prone to be derepressed in *uhrf1* and *dnmt1* mutant livers. Despite the fact that the majority of the TEs were upregulated, we found a group of TEs, mostly DNA transposons, that were downregulated. This surprising finding suggests the existence of compensatory mechanisms that suppress DNA transposons when DNA methylation is removed. This is supported by our previous finding in mouse livers where *uhrf1* loss does not induce TEs expression or cause an immune response, likely due to the relocation H3K27me3 to hypomethylated TEs to compensate for loss of DNA methylation (56). Similar findings were reported in a mouse model of glioblastoma where H3K27 acetylation activates ERVs and that was further enhanced by the global DNA methylation loss in tumors (57). This evidence suggests that repressive histone modifications in collaboration with DNA methylation could control distinct populations of TEs or select those with distinct features, such as their age or CpG content. Further investigation into the repertoire of epigenetic mechanisms that contribute to the distinct patterns of TE expression found here is warranted.

Zebrafish are a powerful and widely used model for studying inflammation and immunity (58). We leveraged these advantages for our studies. In early mouse and zebrafish embryos, *uhrf1* or *dnmt1* is essential for development after gastrulation and depletion or loss of one of these factors leads to early embryonic death (12, 13). Maternal supply of *uhrf1* and *dnmt1* in zebrafish embryos sustains their development through the early stages allowing for examination of embryos at later stages of development. The zebrafish *dnmt1* and *uhrf1* mutants have systemic developmental defects that are revealed at later developmental stages, including reduced size of the digestive organs characterized by an underdeveloped and inflamed gut (8, 16) and a small liver (14, 15, 29). We found that *dnmt1* and *uhrf1* loss induces TEs and activation of anti-viral sensing pathways, culminating in the activation of typeI interferon response and Tnfa signaling. The signaling pathways activated in the liver of these mutants mimic human cells infected with SARS-CoV2 (48), indicating that it is a *bona fide* anti-viral response, including the Tnfa pathway. In hepatocytes, TNFa levels determine the choice between pro-survival or pro-apoptotic signaling as demonstrated in acute liver injury models where TNFa is necessary to protect hepatocytes from apoptosis (59, 60). In other scenarios, Tnfa activation can also promote liver injury (61, 62). Upon DNA methylation loss, we found that *tnfa, tnfr2* and downstream components of the Tnfa signaling pathway were induced (Figure 4E). Importantly, *tnfa* crispants rescued the cell death phenotype in the liver of *uhrf1* mutants (Figure 5C), similarly to what was found in the intestine (8). This could have implications for leveraging this pathway to target cancer cells which show widespread DNA hypomethylation and aberrant TE expression (5, 63).

Despite the growing body of evidence linking the expression of TEs with the activation of anti-viral responses, it is known if the TEs directly cause immune-mediated responses or whether changing the epigenetic landscape that contributes to TE activation also can contribute to the expression of genes involved in inflammation. Some studies interpret the hypomethylation of the *tnfa* promoter in *uhrf1* mutants in both mouse (9) and zebrafish (8) as a finding demonstrating that promoter methylation has a direct role in controlling *tnfa* expression. However, the promoter of *tnfa* in zebrafish, mouse and human does not contain a CpGs island, and indeed in zebrafish it contains only 7 CpGs in the distal promoter (−999 to −620 bp from the transcription start site), indicating that there are only a few potential sites that render this gene susceptible to regulation by DNA methylation. Unfortunately, the RRBS dataset generated here did not cover the *tnfa* promoter with sufficient depth to enable us to examine this locus directly in our samples. However, the preponderance of evidence suggests that DNA methylation is not likely to be a major mechanism regulating *tnfa* but instead an indirect mechanism, mediated by the activation of antiviral sensing pathways causes Tnfa pathway activation. Indeed, our finding shows that deletion of *mavs* and *sting* reduces expression of *tnfa* and significantly reduces the downstream effector, NF-kB. Since there is no expectation that these sensors have any impact on the epigenetic status of the promoter of these genes, we conclude that the most likely explanation for *tnfa* activation in this system is due to response to TE activation. A caveat to our study is that we cannot exclude other possible causes of Tnfa activation or the immune response: for instance, pericentromeric DNA could have been unmasked, *uhrf1* mutants may acquire a different microbiome that changes their immune response or perhaps the loss of *uhrf1* or *dnmt1* in the immune cells makes them more susceptible to immune signaling. Indeed, we cannot exclude that some variable that we have not detected or controlled for is creating the immune response reported in the liver of *uhrf1* and *dnmt1* mutants.

What is the functional relevance of TE activation and induction of the innate immune response? DNA hypomethylation is a common characteristic of cancer cells, and global loss of DNA methylation is found prior to malignant transformation, as the pattern of DNA methylation in senescence cells is the same as those in tumors (64). In this scenario, loss of DNA methylation could lead to expression of TEs, and if these become mobile, they could cause genome instability that is the foundation for cancer cell evolution. We propose that TE expression can be a harbinger of a damaged epigenome, and the resulting immune response can serve to eliminate these damaged and potentially dangerous cells. However, the prolonged activation of an antiviral response can be deleterious as this can promote liver damage, enhance fibrosis and be a key factor promoting tumorigenesis in the liver. Understanding how specific TEs are regulated and defining how inappropriate TEs activation can promote inflammation in the liver will inform the design of tailored approaches that can enhance the aspects of the immune system that repair damage and limit those aspects that promote pathology.

## Data Availability Statement

The datasets presented in this study can be found at: https://www.ncbi.nlm.nih.gov/, GSE160728.

## Ethics Statement

The animal study was reviewed and approved by NYUAD IACUC Committee.

## Author Contributions

EM and KCS conceived the study. EM, FM, BPM, and FA performed the experiments. EM, FM, BPM, CZ, FA, and KCS analyzed the data. EM, FM, BPM, CZ, and KCS wrote the manuscript. All the authors revised the manuscript.

## Conflict of Interest

The authors declare that the research was conducted in the absence of any commercial or financial relationships that could be construed as a potential conflict of interest.
