# Erratum: *uhrf1* and *dnmt1* Loss Induces an Immune Response in Zebrafish Livers Due to Viral Mimicry by Transposable Elements

**DOI:** 10.3389/fimmu.2021.780739

**Published:** 2021-10-11

**Authors:** Frontiers Production Office

**Affiliations:** Frontiers Media SA, Lausanne, Switzerland

**Keywords:** *uhrf1*, *dnmt1*, transposable element, interferon, TNFa, zebrafish, DNA methylation

Due to a production error, there was a mistake in the **Author Contributions** statement. The corrected author contributions statement appears below.

“EM and KCS conceived the study. EM, FM, BPM, and FA performed the experiments. EM, FM, BPM, CZ, FA, and KCS analyzed the data. EM, FM, BPM, CZ, and KCS wrote the manuscript. All the authors revised the manuscript.”

The publisher apologizes for this mistake. The original version of this article has been updated.

