# Supplementary figures and images for: uhrf1 and dnmt1 Loss Induces an Immune Response in Zebrafish Livers Due to Viral Mimicry by Transposable Elements
Source: Front Immunol. 2021 Mar 29;12:627926. doi: 10.3389/fimmu.2021.627926 (PMC8039153; doi:10.3389/fimmu.2021.627926)

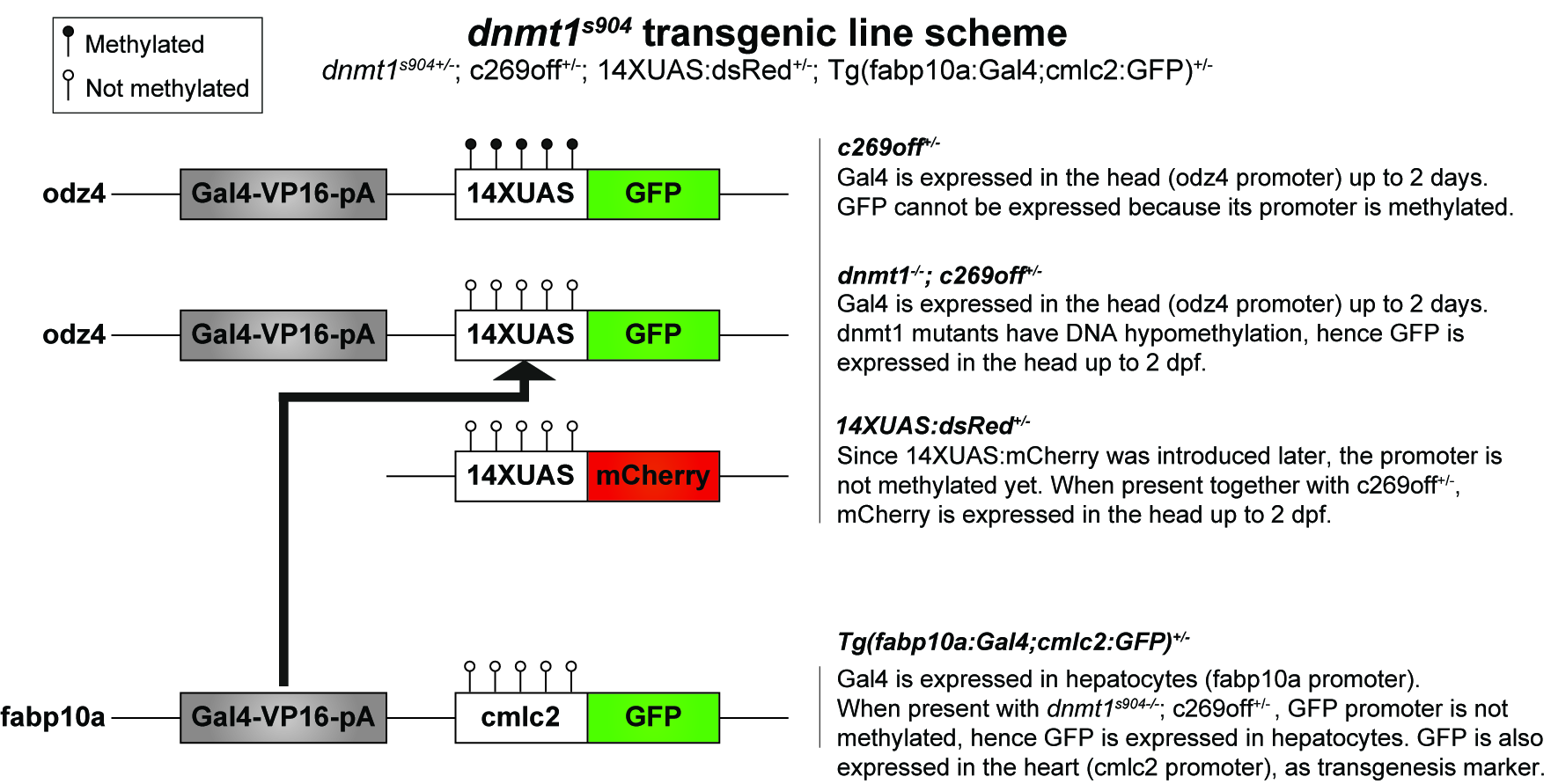

Supplement: Supplementary file 1 [file Image_1.TIF]

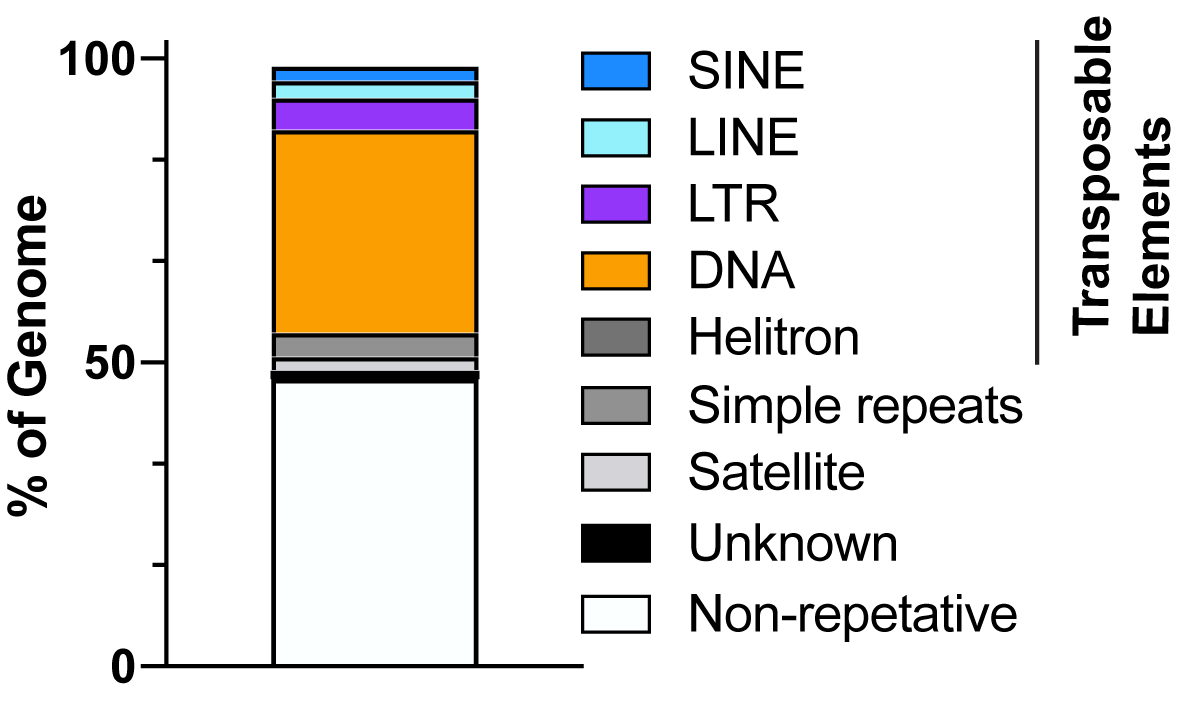

Supplement: Supplementary file 2 [file Image_2.TIF]

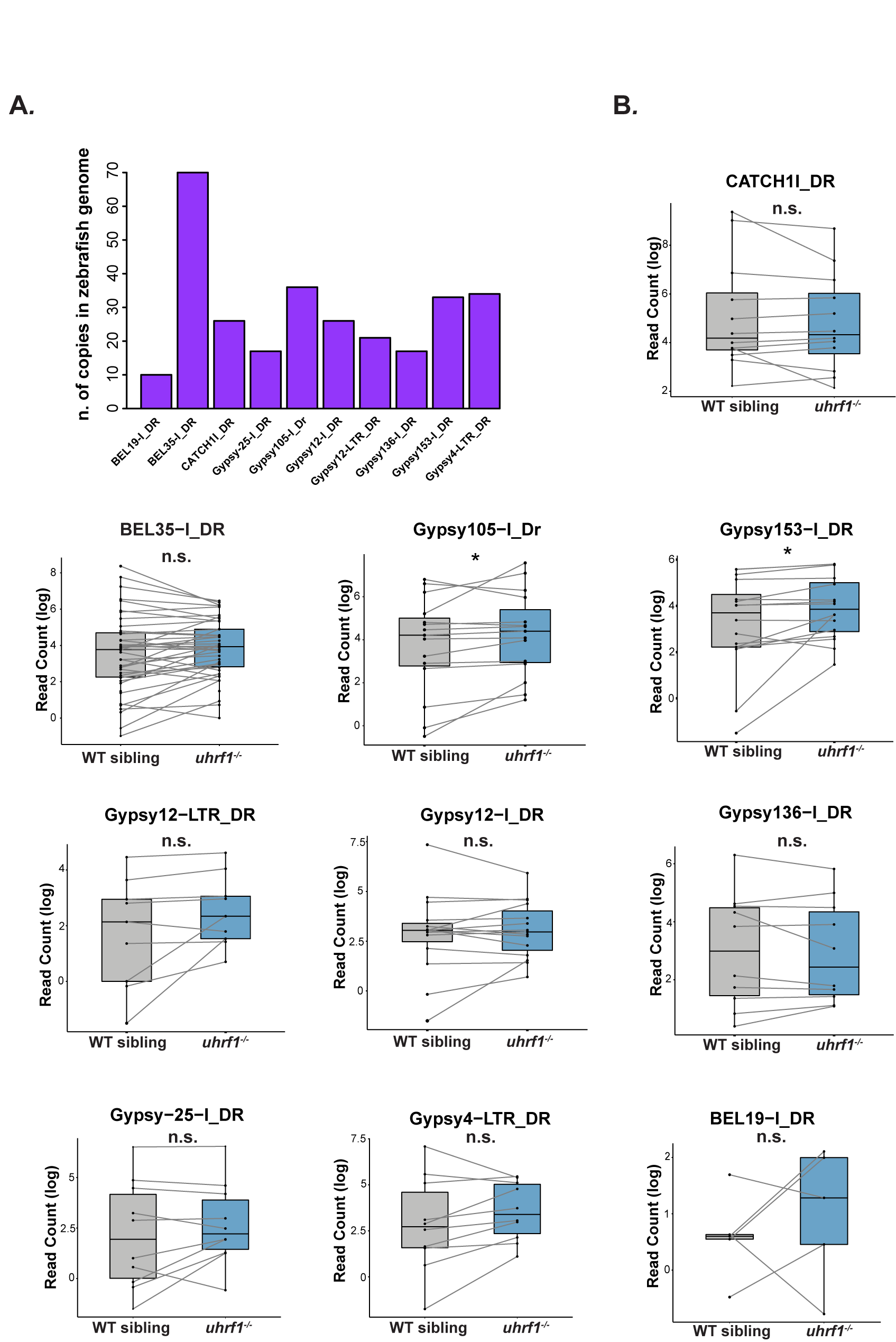

Supplement: Supplementary file 3 [file Image_3.TIF]

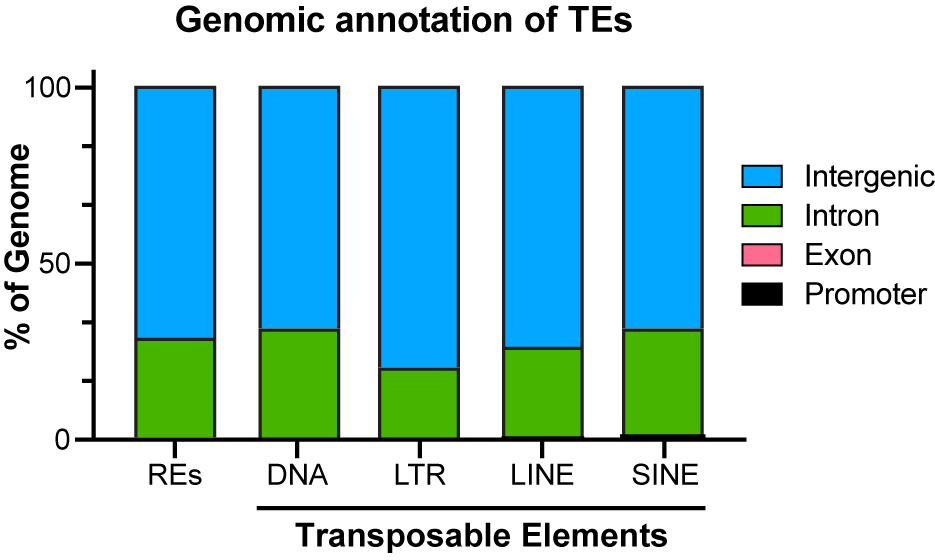

Supplement: Supplementary file 4 [file Image_4.TIF]

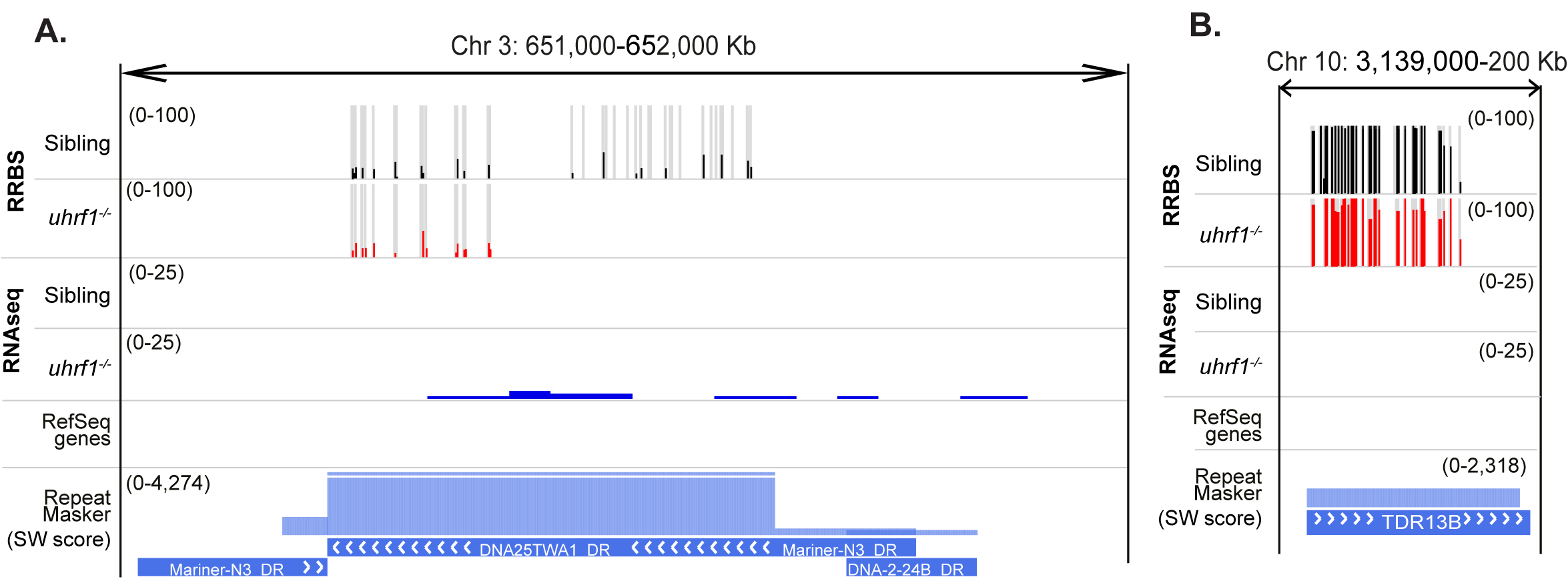

Supplement: Supplementary file 5 [file Image_5.TIF]

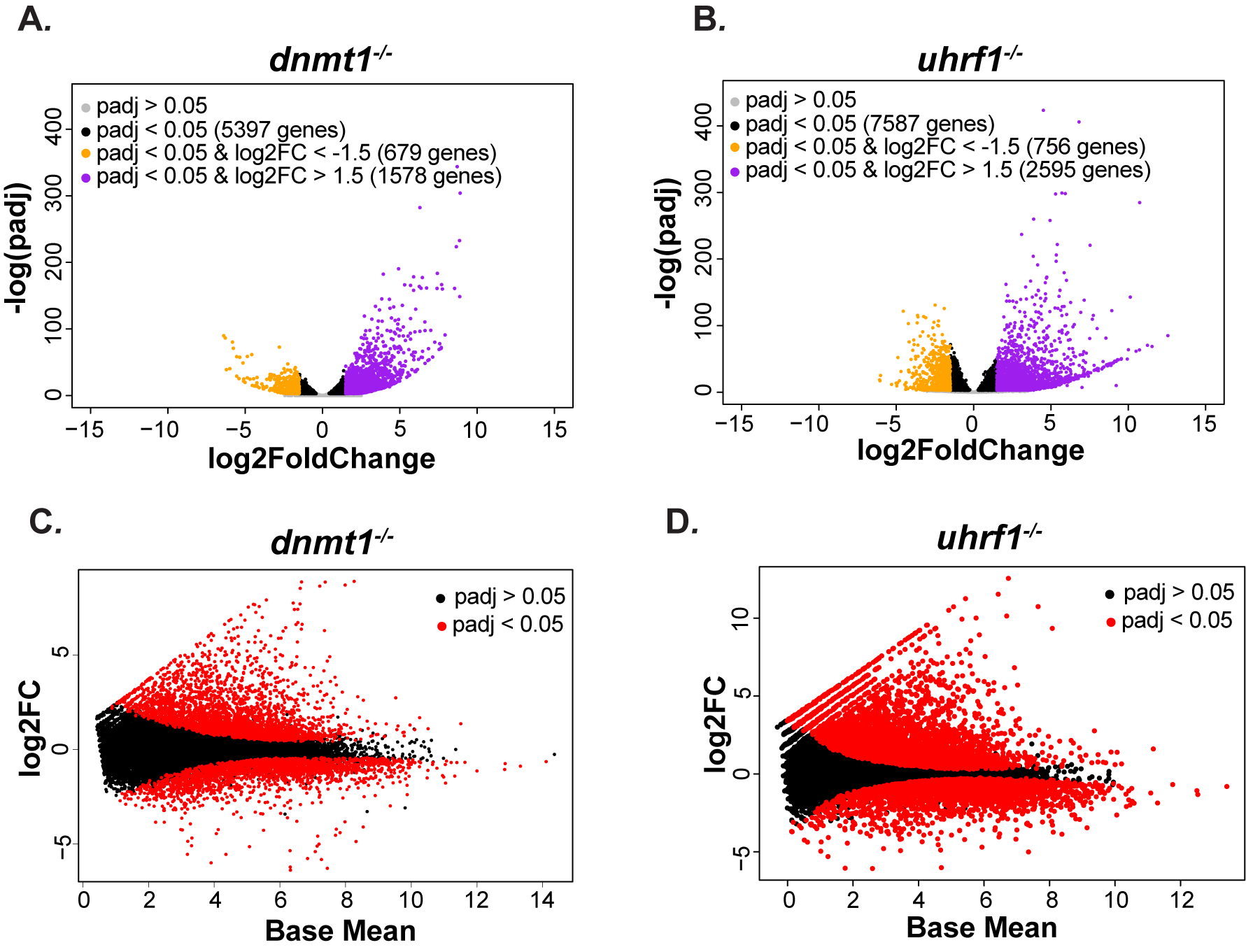

Supplement: Supplementary file 6 [file Image_6.TIF]

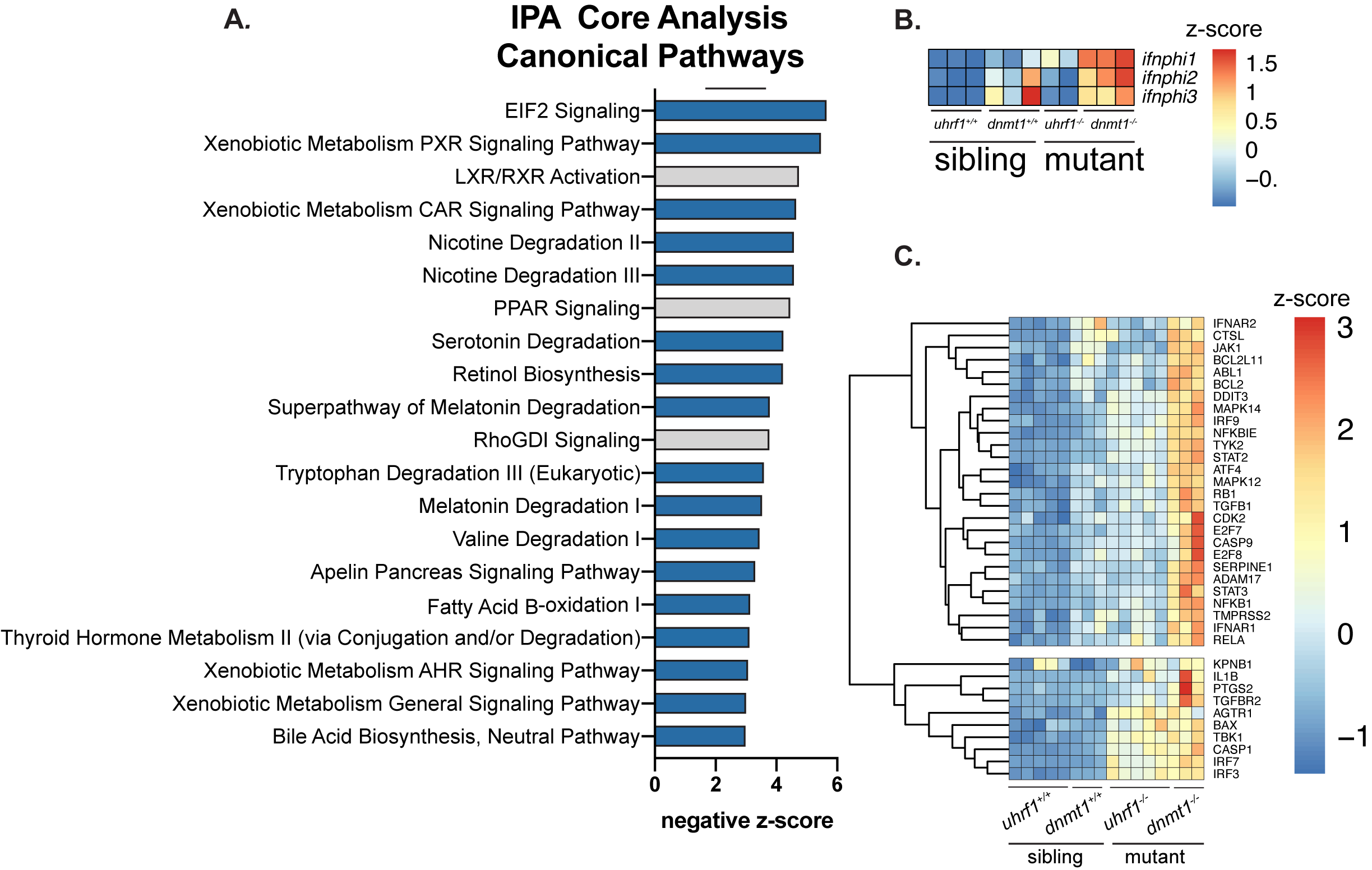

Supplement: Supplementary file 7 [file Image_7.TIF]

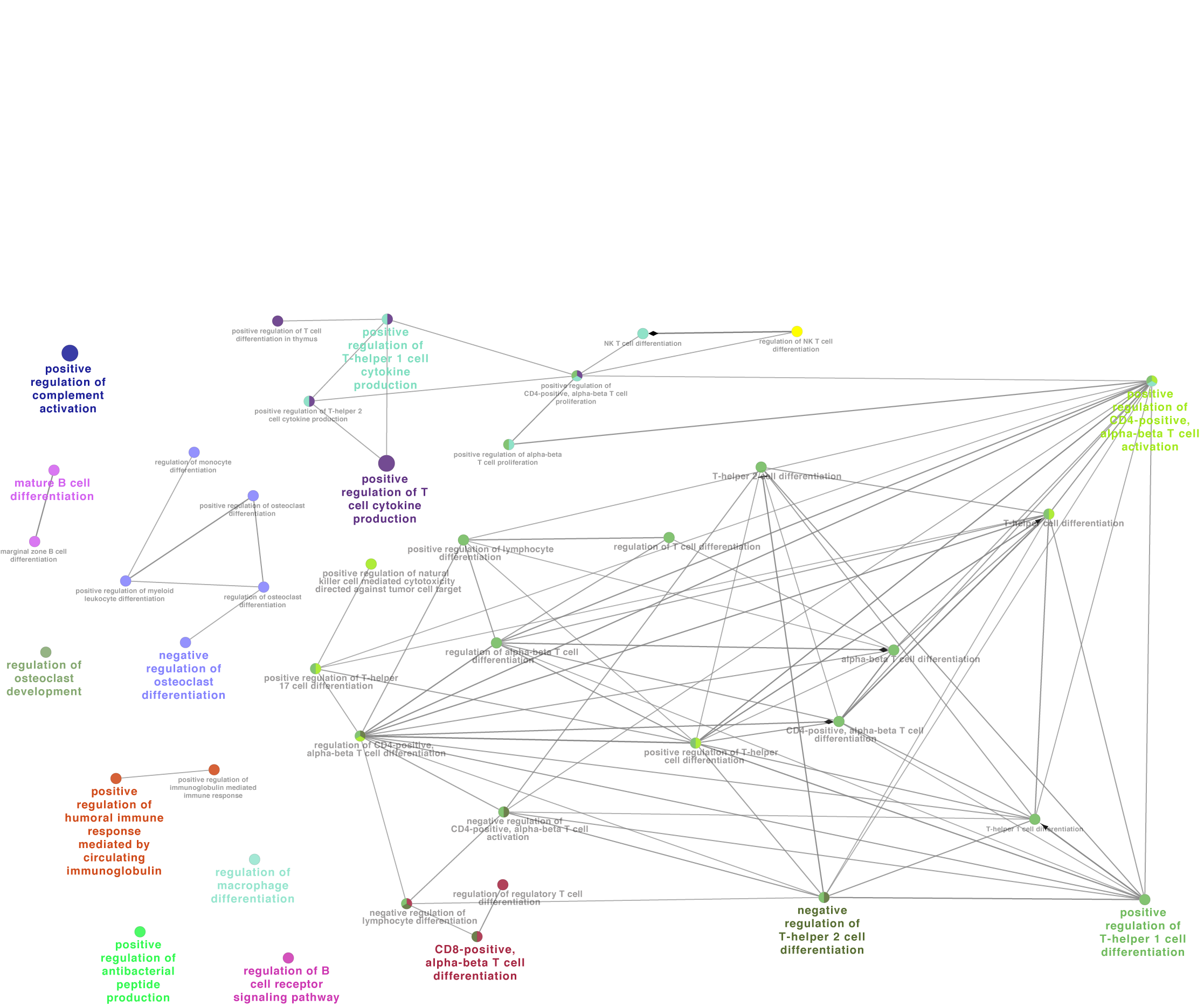

Supplement: Supplementary file 8 [file Image_8.TIF]

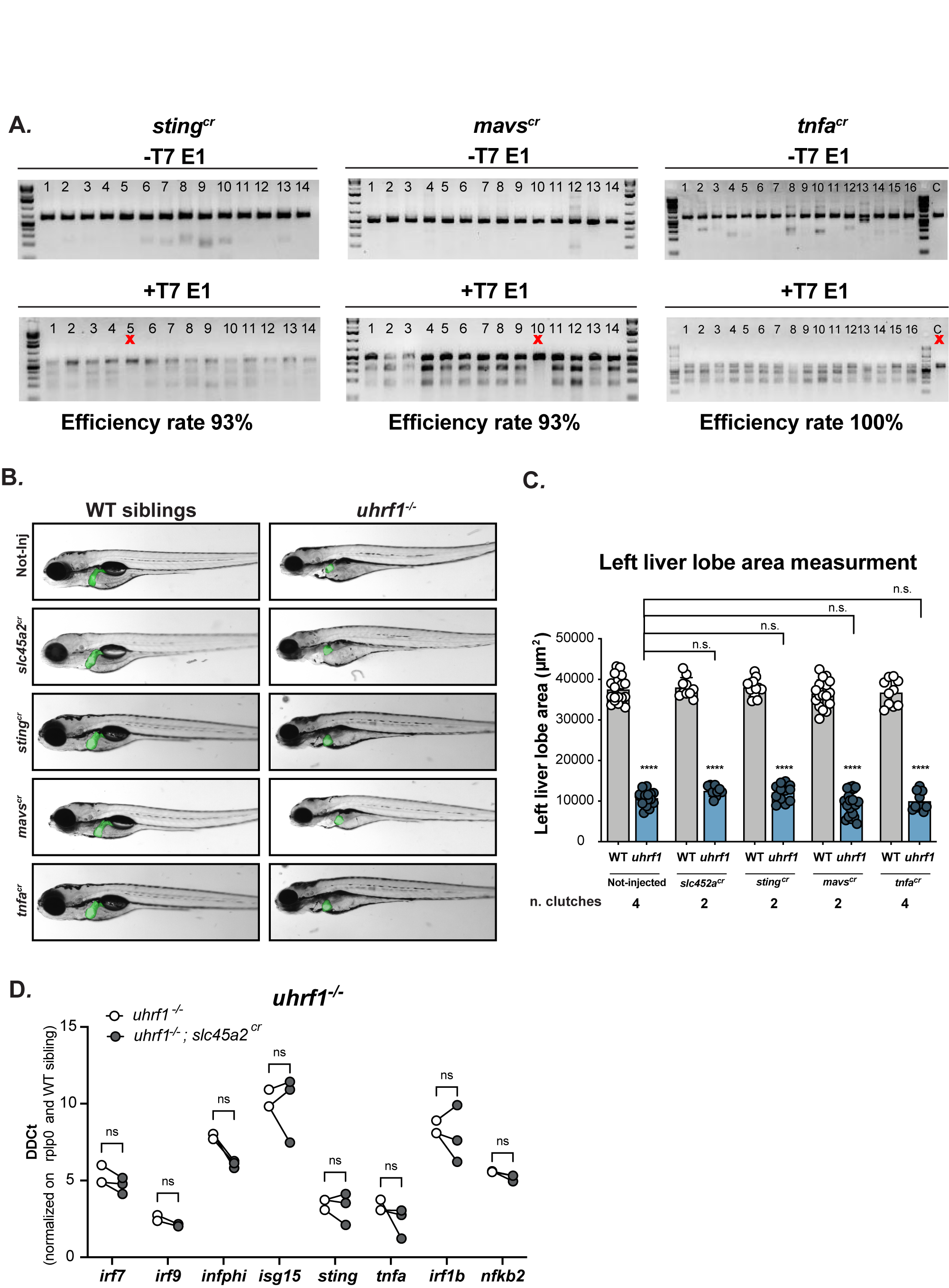

Supplement: Supplementary file 9 [file Image_9.TIF]
