# Supplementary material for: uhrf1 and dnmt1 Loss Induces an Immune Response in Zebrafish Livers Due to Viral Mimicry by Transposable Elements
Source: Front Immunol. 2021 Mar 29;12:627926. doi: 10.3389/fimmu.2021.627926 (PMC8039153; doi:10.3389/fimmu.2021.627926)
Supplement: Supplementary file 14 [file Data_Sheet_1.pdf]

Supplementary Material

Supplemental Materials and Methods

Liver size measurement. Liver size measurements were carried out based on the 2 dimensional area of the left liver lobe in 5 dpf larvae marked with the *Tg(fabp10:CAAX-EGFP)* transgenic line in which the entire liver is marked with EGFP as described previously (Mudbhary et al 2014). Larvae were fixed 4% paraformaldehyde at room temperature for 3 hours, washed with PBS, mounted in 3% methylcellulose on the right side and the left liver lobe was imaged using Nikon Elements on a Nikon SMZ25 Stereoscope. Liver size measurements were calculated in ImageJ using free hand selection of the EFGP labeled area corresponding to the left liver lope, and analyzed using the shape descriptor sub-function to determine the area of the left liver lobe, expressed in μM^2.

Supplementary Figures

Supplemental Figure 1. Detection of DNA hypomethylation in live zebrafish. Schematic representation of the zebrafish line *dnmt1^{s904}*; *c269^{off};14XUAS; (fabp10a:Gal4; cmlc2:GFP).*

Supplemental Figure 2. Repetitive elements are dominated by DNA transposons in the zebrafish genome. Repeat masker was used to annotate the percent of the total genome which is occupied by repetitive (colors) and non-repetitive (white) elements in the genome.

Supplemental Figure 3. Correlation between expression of TEs and nearest genes. A. Copy numbers in the zebrafish reference genome (GRCz10) of the 10 most upregulated TEs in *uhrf1* mutant livers. **B.** For each TE in panel A, the corresponding nearest genes were found and the mean of normalized reads of 3 biological replicates was plotted for *uhrf1* mutant and WT sibling controls. The connected lines show the pairwise expression of each gene in the two samples. Significance is calculated by paired t-test, * means p-value < 0.05.

Supplemental Figure 4. CpGs covered in RRBS are mainly located in intergenic regions. Genomic annotation of CpGs covered in RRBS and overlapping with the TEs annotated in Repeat Masker.

Supplemental Figure 5. Genome browser screenshot of DNA transposons. A. Epigenome Browser (http://epigenomegateway.wustl.edu/browser/) view of the methylation and expression levels on DNA transposon (DNA25TWA1_DR) that is unmethylated and not expressed in WT siblings and stays unmethylated and not expressed in *uhrf1$^{-/-}$* mutants. SW score is determined by Repeat Masker and it is used as indicator of the age of transposons. High SW score corresponds to highly conserved TEs, indicating younger TE. **B.** Epigenome Browser view of the DNA transposons TDR13B that is methylated and not expressed in WT siblings and stays methylated and not expressed in *uhrf1$^{-/-}$* mutants. SW score is determined by Repeat Masker and it is used as indicator of the age of transposons. High SW score corresponds to highly conserved TEs, indicating younger TE.

Supplemental Figure 6. Volcano and MA plot of RNAseq datasets of uhrf1 and dnmt1 mutant livers. A, B. Volcano plot of *dnmt1* and *uhrf1* mutant livers compared to their WT siblings. **C, D.** MAplot of *dnmt1* and *uhrf1* mutant livers compared to their WT siblings.

Supplemental Figure 7. *ifnphi* expression and pathway analysis of down regulated genes in *uhrf1* and *dnmt1* mutant livers. A. Bar graph shows the 20 most down-regulated pathways in both *uhrf1*$^{-/-}$ and *dnmt1*$^{-/-}$ mutants identified by IPA. Pathways are ranked ordered based on z-score and it indicates the likelihood of downregulation states based on comparison with a model that assigns random regulation directions. Pathways in blue are related to primary liver functions such as metabolism. **B.** Heatmap shows the expression of the type I interferon genes in *uhrf1*$^{-/-}$ and *dnmt1*$^{-/-}$ mutants compared to their WT sibling livers. z-score is calculated on read counts of each clutch analyzed by RNAseq. **C.** Heatmap of genes induced upon Coronavirus infection (Fagone et al., 2020). z-score is calculated based on read counts of each clutch.

Supplemental Figure 8. **Immune response is at the center of the regulatory network induced by DNA methylation loss.** ClueGO analysis of all the differentially expressed genes (padj < 0.05) in *uhrf1*$^{-/-}$ and *dnmt1*$^{-/-}$ mutant livers identified an interconnected network of pathways that are dominated by the regulation of immune signaling.

Supplemental Figure 9. Validation of sgRNA for *sting*, *mavs* and *tnfa*. A. T7 Endonuclease I assay has been performed to assess the efficiency of each sgRNAs. When present an indel mutation, T7e1 cut the amplicon by generating smaller fragment as shown in the bottom panel of the sgRNA. Samples that did not show and efficient cut are labeled in red. **B.** Images of *uhrf1* mutant and WT sibling larvae showed no differences in larvae or liver morphology of crispants compared not-injected larvae. **C**. Measurement of left liver lobe area shows no difference between not-injected and injected larvae. Significance is calculated with unpaired t-test. **** means p-value < 0.001. **D**. qPCR of immune genes in livers of larvae injected with *slc45a2* sgRNA (control sgRNA) and not-injected. Significance is calculated with paired t-test.

Supplemental Table 1. List of all the oligonucleotides used in this publication.

Supplemental Table 2. List of upregulated TEs in *uhrf1* and *dnmt1* mutant livers with full information regarding Families and Class.

Supplemental Table 3. RNAseq data for genes identified as differentially expressed and involved in the interferon pathway by IPA analysis. The IPA pathway diagram showing relative expression of these genes in *uhrf1*$^{-/-}$ and *dnmt1*$^{-/-}$ mutant livers compared to their respective sibling controls is displayed in Figure 4D.

Supplemental Table 4. RNAseq data for genes identified as differentially expressed and involved in the Tnf pathway by IPA analysis. The IPA pathway diagram showing relative expression of these genes in *uhrf1*$^{-/-}$ and *dnmt1*$^{-/-}$ mutant livers compared to their respective sibling controls is displayed in Figure E.
